# Supplementary material for: Variable expression quantitative trait loci analysis of breast cancer risk variants
Source: Sci Rep. 2021 Mar 30;11:7192. doi: 10.1038/s41598-021-86690-5 (PMC8009949; doi:10.1038/s41598-021-86690-5)
Supplement: Supplementary file 1 — Supplementary Information [file 41598_2021_86690_MOESM1_ESM.pdf]

Variable expression quantitative trait loci analysis of breast cancer risk variants

Wiggins, George A R[1], Black, Michael A[2], Dunbier, Anita[2], Merriman, Tony R[2], Pearson, John F[1,3#], Walker, Logan C[1#*]

[1]*Department of Pathology and Biomedical Science, University of Otago Christchurch, NZ.*

[2]*Department of Biochemistry, University of Otago Dunedin, NZ.*

[3]*Biostatistics and Computational Biology Unit, University of Otago Christchurch, NZ.*

[#]*Contributed equally*

[*]*Corresponding author:* Logan C. Walker

Tel: +64 3 364 0544

Email: logan.walker@otago.ac.nz

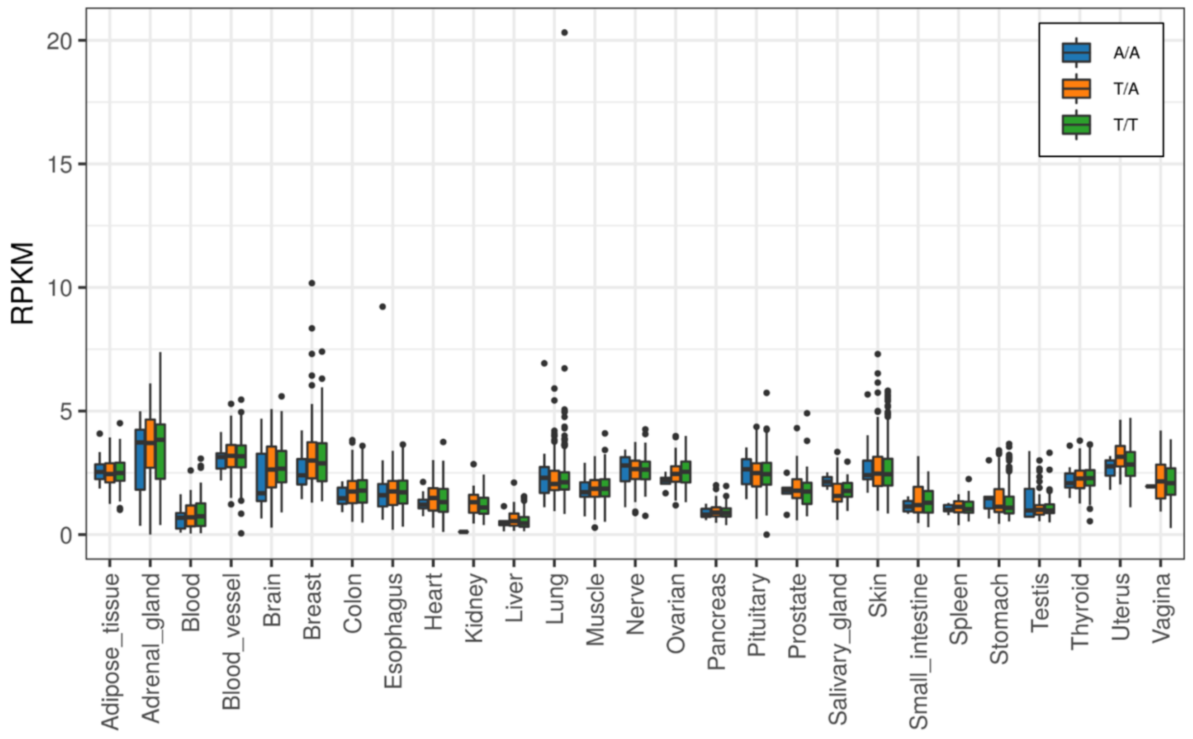

Supplementary Figure S1. Tissue specific expression of IRX3 stratified by genotypes at the rs11075995 location. T/T homozygous major allele (Green), A/T heterozygous (Orange), A/A homozygous minor allele (Blue).

Supplementary Table S1. Known variants associated with breast cancer risk

				Allele counts: Breast				Allele counts: Kidney				Allele counts: Lung				Allele counts: Ovary			
Chromosomal location	Allele	Risk variants	Reference	AA	Aa	aa	No call	AA	Aa	aa	No call	AA	Aa	aa	No call	AA	Aa	aa	No call
1_10566215	A/G	rs616488	Michailidou, K. et al. (2013)	129	108	18	0	19	19	4	0	196	161	31	0	68	47	8	0
1_18807339	T/C	rs2992756	Michailidou, K et al. (2017)	60	135	60	0	11	22	9	0	106	189	92	1	35	63	25	0
1_41380440	C/T	rs4233486	Michailidou, K et al. (2017)	41	108	106	0	7	21	14	0	51	171	166	0	13	58	52	0
1_42137311	T/G	rs79724016	Michailidou, K et al. (2017)	243	12	0	0	39	3	0	0	361	27	0	0	120	3	0	0
1_46600917	A/G/T	rs1707302	Michailidou, K et al. (2017)	20	132	103	0	3	19	20	0	33	184	171	0	12	66	45	0
1_50846033	AAAGGGCAAGATCTCCTTTTT/-	rs140850326	Michailidou, K et al. (2017)	NA	NA	NA	NA	NA	NA	NA	NA	NA	NA	NA	NA	NA	NA	NA	NA
1_88156923	G/A	rs17426269	Michailidou, K et al. (2017)	194	54	6	1	35	7	0	0	296	82	9	1	99	21	2	1
1_114448389	C/T	rs11552449	Michailidou, K. et al. (2013)	176	72	7	0	33	8	1	0	283	91	14	0	87	34	2	0
1_118230221	T/C	rs7529522	Michailidou, K et al. (2017)	127	108	20	0	18	20	4	0	210	149	29	0	67	48	8	0
1_121280613	A/C/G	rs11249433	Thomas, G. et al. (2009)	109	102	43	1	21	16	5	0	170	158	59	1	63	52	8	0
1_145644984	C/T	rs12405132	Finucane, H. K. et al. (2015)	127	104	24	0	21	19	2	0	187	156	44	1	56	53	13	1
1_149927034	A/C	rs12048493	Finucane, H. K. et al. (2015)	122	103	27	3	18	20	4	0	180	157	45	6	62	50	10	1
1_155148781	G/A	rs4971059	Michailidou, K et al. (2017)	119	106	30	0	15	21	6	0	172	169	47	0	58	51	14	0
1_201437832	C/T	rs35383942	Michailidou, K et al. (2017)	235	20	0	0	35	7	0	0	350	37	1	0	111	12	0	0
1_202187176	G/A	rs6678914	Garcia- Closas, M. et al. (2013)	75	143	37	0	15	21	6	0	126	198	64	0	40	59	24	0
1_203766331	A/G	rs4951011	Cai, Q. et al. (2014)	169	83	3	0	32	6	4	0	270	110	8	0	89	32	2	0
1_204518842	C/A/G	rs4245739	Garcia- Closas, M. et al. (2013)	16	88	151	0	2	10	30	0	30	140	218	0	4	41	78	0
1_217220574	G/A	rs11117758	Michailidou, K et al. (2017)	163	83	9	0	28	10	4	0	247	126	15	0	81	37	5	0
1_242034263	A/G	rs72755295	Finucane, H. K. et al. (2015)	251	4	0	0	39	3	0	0	373	15	0	0	121	2	0	0
2_10135681	C/G	rs113577745	Michailidou, K et al. (2017)	199	53	1	2	34	8	0	0	314	70	1	3	96	26	0	1
2_19320803	T/A/C	rs12710696	Garcia- Closas, M. et al. (2013)	43	111	101	0	1	22	19	0	59	180	149	0	14	60	49	0
2_24739695	-/T/TT/TTT	rs200648189	Milne et al. (2017)	NA	NA	NA	NA	NA	NA	NA	NA	NA	NA	NA	NA	NA	NA	NA	NA
2_25129473	A/G	rs6725517	Michailidou, K et al. (2017)	70	122	63	0	17	14	11	0	114	183	91	0	29	58	36	0
2_29120733	C/T	rs4577244	Couch, F. J. et al. (2016)	151	90	13	1	23	17	2	0	227	141	18	2	72	48	2	1
2_111925738	TTTATGT/-	rs71801447	Michailidou, K et al. (2017)	NA	NA	NA	NA	NA	NA	NA	NA	NA	NA	NA	NA	NA	NA	NA	NA
2_121245122	T/A/C	rs4849887	Michailidou, K. et al. (2013)	9	54	192	0	0	9	33	0	10	80	298	0	6	26	91	0
2_172972971	G/A	rs2016394	Michailidou, K. et al. (2013)	84	122	49	0	15	18	9	0	132	182	74	0	40	58	25	0
2_174212894	G/A	rs1550623	Michailidou, K. et al. (2013)	7	73	175	0	1	15	26	0	7	93	288	0	1	36	86	0
2_202181247	C/T	rs1830298	Lin, W. Y. et al. (2015)	23	90	140	2	1	16	24	1	26	145	216	1	13	42	68	0
2_217920769	G/T	rs4442975	Ghoussaini, M. et al. (2014)	79	117	59	0	12	23	7	0	117	186	85	0	37	52	34	0
2_217963060	C/A/G/T	rs34005590	Wyszynski, A. et al. (2016)	232	22	1	0	36	6	0	0	350	36	2	0	110	13	0	0
2_218296508	C/T	rs16857609	Michailidou, K. et al. (2013)	138	95	22	0	27	12	3	0	220	141	26	1	67	48	7	1
2_227226952	A/G	rs12479355	Michailidou, K et al. (2017)	162	81	12	0	27	13	2	0	237	133	18	0	78	38	7	0
3_4742276	A/G	rs6762644	Michailidou, K. et al. (2013)	84	138	33	0	15	20	7	0	139	193	56	0	42	61	20	0
3_27416013	C/T	rs4973768	Ahmed, S. et al. (2009)	77	134	42	2	11	26	4	1	116	197	71	4	39	65	17	2
3_30682939	G/C/T	rs12493607	Michailidou, K. et al. (2013)	103	118	34	0	22	15	5	0	154	185	49	0	61	55	7	0
3_46866866	G/A/T	rs6796502	Finucane, H. K. et al. (2015)	209	45	1	0	38	4	0	0	319	66	3	0	96	25	2	0
3_63967900	A/G	rs1053338	Milne, R. L. et al. (2014)	179	72	4	0	32	8	2	0	272	108	8	0	94	27	2	0
3_71532113	T/A/C/G	rs6805189	Michailidou, K et al. (2017)	79	135	41	0	11	21	10	0	121	191	75	1	26	70	27	0
3_87037543	A/G	rs13066793	Michailidou, K et al. (2017)	214	38	0	3	34	8	0	0	326	56	2	4	100	21	0	2
3_99723580	G/T	rs9833888	Michailidou, K et al. (2017)	168	70	17	0	26	14	2	0	261	110	17	0	81	32	10	0
3_141112870	TT/-	rs34207738	Michailidou, K et al. (2017)	NA	NA	NA	NA	NA	NA	NA	NA	NA	NA	NA	NA	NA	NA	NA	NA
3_172285237	G/A	rs58058861	Michailidou, K et al. (2017)	150	89	15	1	25	14	3	0	247	117	23	1	81	37	5	0
4_38816338	A/C	rs6815814	Michailidou, K et al. (2017)	138	83	34	0	23	10	9	0	201	137	50	0	60	46	17	0
4_84370124	A/-	rs10718573	Michailidou, K et al. (2017)	NA	NA	NA	NA	NA	NA	NA	NA	NA	NA	NA	NA	NA	NA	NA	NA
4_89243818	C/T	rs10022462	Michailidou, K et al. (2017)	87	126	42	0	10	22	10	0	119	198	70	1	49	54	20	0
4_106084778	C/T	rs9790517	Michailidou, K. et al. (2013)	157	91	7	0	25	16	1	0	243	133	12	0	82	37	4	0
4_126843504	G/T	rs77528541	Michailidou, K et al. (2017)	193	50	1	11	31	10	0	1	296	74	6	12	95	22	1	5
4_175846426	C/A	rs6828523	Michailidou, K. et al. (2013)	175	73	7	0	29	10	3	0	290	88	9	1	82	37	4	0
5_345109	T/C	rs116095464	Michailidou, K et al. (2017)	NA	NA	NA	NA	NA	NA	NA	NA	NA	NA	NA	NA	NA	NA	NA	NA
5_1279790	C/T	rs10069690	Haiman, C. A. et al. (2011)	136	100	19	0	18	19	5	0	201	157	30	0	55	56	12	0
5_1296256	-/G	rs3215401	Bojesen, S. E. et al. (2013)	NA	NA	NA	NA	NA	NA	NA	NA	NA	NA	NA	NA	NA	NA	NA	NA
5_16187528	G/A/C/T	rs13162653	Finucane, H. K. et al. (2015)	91	123	40	1	15	21	6	0	131	190	67	0	43	60	20	0
5_32567732	C/T	rs2012709	Finucane, H. K. et al. (2015)	100	109	46	0	17	14	11	0	131	184	73	0	40	60	23	0
5_44706498	A/G	rs10941679	Ghoussaini, M. et al. (2016)	138	101	14	2	19	20	2	1	208	155	22	3	66	46	9	2
5_49641645	T/C	rs72749841	Michailidou, K et al. (2017)	189	60	6	0	29	13	0	0	284	94	8	2	80	37	5	1
5_50195094	-/T/TT/TTT	rs35951924	Michailidou, K et al. (2017)	NA	NA	NA	NA	NA	NA	NA	NA	NA	NA	NA	NA	NA	NA	NA	NA
5_56053723	A/T	rs62355902	Glubb, D. M. et al. (2015)	183	63	9	0	35	6	1	0	265	115	8	0	83	35	5	0
5_58184061	T/A/C	rs10472076	Michailidou, K. et al. (2013)	106	121	28	0	15	24	3	0	159	174	55	0	58	49	16	0
5_58337481	T/G	rs1353747	Michailidou, K. et al. (2013)	210	41	4	0	38	4	0	0	330	52	6	0	99	21	3	0
5_81538046	T/A	rs7707921	Finucane, H. K. et al. (2015)	9	85	160	1	2	6	34	0	16	135	236	1	9	35	79	0
5_90732225	C/T	rs10474352	Cai, Q. et al. (2014)	163	80	12	0	28	11	3	0	252	120	16	0	71	47	5	0
5_111217786	G/T	rs6882649	Michailidou, K et al. (2017)	40	111	104	0	6	20	16	0	64	152	172	0	24	52	47	0
5_132407058	C/T	rs6596100	Michailidou, K et al. (2017)	157	86	12	0	28	14	0	0	237	132	19	0	75	37	11	0
5_158244083	C/T	rs1432679	Michailidou, K. et al. (2013)	68	121	66	0	13	19	10	0	97	182	109	0	37	50	36	0
5_169591487	G/T	rs4562056	Michailidou, K et al. (2017)	127	109	19	0	21	15	6	0	185	165	38	0	57	55	11	0
6_1318878	C/T	rs11242675	Michailidou, K. et al. (2013)	43	120	92	0	3	18	21	0	63	177	148	0	21	53	49	0
6_10456706	C/A	rs9348512	Gaudet, M. M. et al. (2013)	115	113	27	0	14	22	6	0	170	169	49	0	54	59	10	0
6_13722523	G/A	rs204247	Michailidou, K. et al. (2013)	48	139	68	0	7	21	14	0	78	195	115	0	23	62	38	0
6_16399557	C/T	rs3819405	Michailidou, K et al. (2017)	107	117	30	1	18	21	3	0	177	173	37	1	55	56	12	0
6_20621238	T/C	rs2223621	Michailidou, K et al. (2017)	31	118	106	0	4	15	23	0	47	184	155	2	17	55	50	1
6_26680698	G/A	rs71557345	Michailidou, K et al. (2017)	228	20	0	7	36	4	0	2	341	38	0	9	109	12	0	2
6_28926220	G/A/C/T	rs9257408	Finucane, H. K. et al. (2015)	NA	NA	NA	NA	NA	NA	NA	NA	NA	NA	NA	NA	NA	NA	NA	NA
6_81094287	G/A	rs12207986	Michailidou, K et al. (2017)	84	114	54	3	14	17	10	1	131	154	100	3	42	54	27	0
6_82128386	T/C	rs17529111	Siddiq, A. et al. (2012)	159	81	10	5	30	10	1	1	238	130	15	5	76	41	4	2
6_130349119	C/T	rs6569648	Michailidou, K et al. (2017), Milne et al (2017)	12	80	163	0	6	14	22	0	25	123	237	3	6	38	77	2
6_149608874	G/A	rs9485372	Long, J. et al. (2012)	158	86	11	0	33	7	2	0	248	123	16	1	75	42	6	0
6_151942194	T/G	rs3757322	Dunning, A. M. et al. (2016)	120	103	31	1	17	20	4	1	171	167	48	2	47	58	17	1
6_151952332	G/A	rs9397437	Dunning, A. M. et al. (2016)	216	35	3	1	40	1	0	1	337	46	2	3	99	20	2	2
6_152437016	T/C	rs2747652	Dunning, A. M. et al. (2016)	66	126	63	0	9	25	8	0	89	198	101	0	24	68	31	0
7_21940960	A/G	rs7971	Michailidou, K et al. (2017)	NA	NA	NA	NA	NA	NA	NA	NA	NA	NA	NA	NA	NA	NA	NA	NA
7_28356889	T/C/G	rs17156577	Michailidou, K et al. (2017)	187	63	5	0	23	17	2	0	280	97	11	0	87	32	4	0
7_91630620	G/T	rs6964587	Milne, R. L. et al. (2014)	87	135	30	3	16	14	10	2	139	184	61	4	45	57	18	3
7_94113799	T/C	rs17268829	Michailidou, K et al. (2017)	139	101	14	1	27	15	0	0	223	138	26	1	72	45	5	1
7_101552440	G/A	rs71559437	Michailidou, K et al. (2017)	206	47	1	1	36	6	0	0	301	81	2	4	106	16	1	0
7_130667121	C/T	rs4593472	Finucane, H. K. et al. (2015)	99	121	35	0	20	19	3	0	158	177	53	0	56	54	13	0
7_139942304	G/A	rs11977670	Sawyer, E. et al. (2014)	76	133	46	0	13	16	13	0	122	194	72	0	41	59	23	0
7_144074929	G/A	rs720475	Michailidou, K. et al. (2013)	147	96	12	0	30	10	2	0	233	134	21	0	74	43	6	0
8_170692	T/A/C	rs66823261	Milne et al. (2017)	153	87	15	0	27	12	3	0	235	127	26	0	79	36	8	0
8_29509616	A/C	rs9693444	Michailidou, K. et al. (2013)	23	120	112	0	6	16	20	0	38	170	180	0	12	60	51	0
8_36858483	A/G	rs13365225	Finucane, H. K. et al. (2015)	159	85	11	0	25	14	3	0	243	123	22	0	75	42	6	0
8_76230301	G/T	rs6472903	Michailidou, K. et al. (2013)	3	66	186	0	1	14	26	1	8	103	276	1	4	35	83	1
8_76417937	A/G	rs2943559	Michailidou, K. et al. (2013)	214	35	1	5	35	5	0	2	315	62	2	9	93	24	1	5
8_102478959	A/G/T	rs514192	Michailidou, K et al. (2017)	31	111	113	0	5	18	19	0	38	177	173	0	14	55	54	0
8_106358620	A/T	rs12546444	Michailidou, K et al. (2017)	NA	NA	NA	NA	NA	NA	NA	NA	NA	NA	NA	NA	NA	NA	NA	NA
8_117209548	A/G	rs13267382	Finucane, H. K. et al. (2015)	58	103	94	0	10	19	13	0	85	167	136	0	30	56	37	0
8_124610166	G/A/C	rs58847541	Michailidou, K et al. (2017)	182	65	8	0	27	15	0	0	264	114	10	0	81	38	4	0
8_124757661	C/T	rs17350191	Milne et al. (2017)	124	104	27	0	19	19	4	0	201	141	46	0	63	48	12	0
8_128355618	A/G	rs13281615	Easton, D. F. et al. (2007)	88	121	43	3	10	20	12	0	128	185	72	3	42	57	24	0
8_129194641	C/T	rs11780156	Michailidou, K. et al. (2013)	180	67	8	0	33	9	0	0	273	104	11	0	93	24	6	0
9_22062134	G/T	rs1011970	Turnbull, C. et al. (2010)	172	71	11	1	28	13	1	0	273	102	13	0	77	39	6	1
9_110306115	C/A/T	rs10759243	Michailidou, K. et al. (2013)	118	104	33	0	18	19	5	0	182	156	50	0	62	47	14	0
9_110837073	A/G	rs10816625	Orr, N. et al. (2015)	221	30	4	0	36	6	0	0	332	51	5	0	106	15	2	0
9_110837176	C/T	rs13294895	Orr, N. et al. (2015)	198	53	4	0	33	9	0	0	286	88	13	1	95	28	0	0
9_110895353	C/T	rs676256	Orr, N. et al. (2015)	32	117	106	0	4	22	16	0	55	177	156	0	10	55	58	0
9_119313486	A/G	rs1895062	Michailidou, K et al. (2017)	116	105	34	0	16	21	5	0	160	171	57	0	61	49	13	0
9_129396434	G/A	rs10760444	Michailidou, K et al. (2017)	62	122	71	0	6	19	17	0	83	191	114	0	27	57	39	0
9_136151580	GGTGCAGGCGCAGGAAAAAATTGT	rs8176636	Michailidou, K et al. (2017)	NA	NA	NA	NA	NA	NA	NA	NA	NA	NA	NA	NA	NA	NA	NA	NA
10_5886734	C/T	rs2380205	Turnbull, C. et al. (2010)	85	121	49	0	19	16	7	0	129	173	86	0	33	68	22	0
10_9088114	G/-	rs67958007	Michailidou, K et al. (2017)	NA	NA	NA	NA	NA	NA	NA	NA	NA	NA	NA	NA	NA	NA	NA	NA
10_22032942	A/G	rs7072776	Michailidou, K. et al. (2013)	19	101	135	0	4	21	17	0	35	173	180	0	8	62	53	0
10_22315843	A/C	rs11814448	Michailidou, K. et al. (2013)	210	28	15	2	35	3	4	0	322	44	16	6	100	14	9	0
10_64299890	A/G	rs10995201	Darabi, H. et al. (2015)	175	73	2	5	30	12	0	0	266	111	7	4	86	32	4	1
10_80841148	T/C	rs704010	Turnbull, C. et al. (2010)	37	114	104	0	5	14	23	0	50	191	147	0	17	49	57	0
10_95292188	AA/-	rs140936696	Michailidou, K et al. (2017)	NA	NA	NA	NA	NA	NA	NA	NA	NA	NA	NA	NA	NA	NA	NA	NA
10_114773927	A/G	rs7904519	Michailidou, K. et al. (2013)	62	125	68	0	7	16	19	0	97	185	106	0	26	65	32	0
10_123093901	C/T	rs11199914	Michailidou, K. et al. (2013)	109	110	36	0	17	19	6	0	163	171	54	0	43	57	23	0
10_123340311	C/A/T	rs2981578	Meyer, K. B. et al. (2013)	75	122	58	0	9	25	8	0	106	190	92	0	35	57	31	0
10_123340432	C/-	rs35054928	Meyer, K. B. et al. (2013)	NA	NA	NA	NA	NA	NA	NA	NA	NA	NA	NA	NA	NA	NA	NA	NA
10_123349324	A/T	rs45631563	Meyer, K. B. et al. (2013)	239	15	1	0	38	4	0	0	353	34	1	0	116	6	1	0
11_803017	A/G	rs6597981	Michailidou, K et al. (2017)	93	112	50	0	14	20	8	0	128	172	88	0	45	49	29	0
11_1909006	T/C	rs3817198	Easton, D. F. et al. (2007)	130	99	26	0	23	16	3	0	191	159	38	0	61	52	10	0
11_65583066	G/T	rs3903072	Michailidou, K. et al. (2013)	85	118	52	0	16	19	7	0	107	195	86	0	43	57	23	0
11_69331642	C/A/G/T	rs554219	French, J. D. et al. (2013)	184	65	6	0	28	11	3	0	291	91	6	0	92	28	3	0
11_69379161	C/A	rs75915166	French, J. D. et al. (2013)	224	30	1	0	38	4	0	0	344	44	0	0	112	11	0	0
11_108345516	-/A	rs11374964	Milne et al. (2017)	NA	NA	NA	NA	NA	NA	NA	NA	NA	NA	NA	NA	NA	NA	NA	NA
11_108357137	G/A	rs74911261	Milne et al. (2017)	247	8	0	0	38	4	0	0	372	16	0	0	119	4	0	0
11_129461171	T/C/G	rs11820646	Michailidou, K. et al. (2013)	37	113	105	0	6	15	21	0	58	184	146	0	20	51	52	0
12_14413931	G/C	rs12422552	Michailidou, K. et al. (2013)	122	110	23	0	22	14	6	0	196	165	27	0	58	56	9	0
12_28174817	C/T	rs7297051	Zeng, C. et al. (2016)	144	98	13	0	21	20	1	0	224	143	20	1	66	49	8	0
12_85009437	T/C	rs202049448	Michailidou, K et al. (2017)	NA	NA	NA	NA	NA	NA	NA	NA	NA	NA	NA	NA	NA	NA	NA	NA
12_96027759	A/G	rs17356907	Michailidou, K. et al. (2013)	132	106	17	0	20	14	8	0	208	149	31	0	74	39	10	0
12_115836522	A/G	rs1292011	Ghoussaini, M. et al. (2012)	87	125	43	0	15	15	12	0	119	194	75	0	39	65	19	0
12_120832146	C/T	rs206966	Michailidou, K et al. (2017)	193	59	3	0	32	9	1	0	286	96	6	0	90	31	2	0
13_32972626	A/T	rs11571833	Michailidou, K. et al. (2013)	252	3	0	0	42	0	0	0	379	9	0	0	121	2	0	0
13_73957681	A/G	rs6562760	Couch, F. J. et al. (2016)	18	99	137	1	2	15	25	0	24	142	222	0	7	42	74	0
14_37132769	G/A/C	rs2236007	Michailidou, K. et al. (2013)	169	76	10	0	34	7	1	0	254	119	15	0	85	31	7	0
14_68660428	T/C	rs2588809	Michailidou, K. et al. (2013)	7	80	167	1	3	13	26	0	14	107	265	2	6	36	80	1
14_69034682	C/T	rs999737	Thomas, G. et al. (2009)	166	75	14	0	31	8	3	0	240	131	17	0	77	41	5	0
14_91841069	A/G	rs941764	Michailidou, K. et al. (2013)	91	125	39	0	17	21	4	0	167	168	53	0	45	56	22	0
14_93104072	T/C	rs11627032	Finucane, H. K. et al. (2015)	141	93	21	0	14	24	4	0	219	147	22	0	79	39	5	0
14_105212262	-/TT	rs10623258	Michailidou, K et al. (2017)	NA	NA	NA	NA	NA	NA	NA	NA	NA	NA	NA	NA	NA	NA	NA	NA
15_91512067	G/A	rs2290203	Cai, Q. et al. (2014)	146	94	15	0	28	12	1	1	223	129	35	1	67	45	11	0
16_4106788	C/A/T	rs11076805	Milne et al. (2017)	153	85	17	0	24	14	4	0	233	124	31	0	76	40	7	0
16_52599188	C/T	rs4784227	Ghoussaini, M. et al. (2012)	154	93	8	0	24	18	0	0	224	149	15	0	73	48	2	0
16_53813367	T/A/G	rs17817449	Michailidou, K. et al. (2013)	97	124	34	0	15	20	7	0	142	197	49	0	47	58	18	0

16_53855291	A/T	rs11075995	Garcia- Closas, M. et al. (2013)	11	86	158	0	1	14	27	0	13	147	228	0	4	46	73	0
16_54682064	G/A	rs28539243	Michailidou, K et al. (2017)	56	126	73	0	8	13	21	0	84	185	119	0	26	55	42	0
16_56420987	A/C/G	rs2432539	Michailidou, K et al. (2017)	26	113	108	8	6	12	23	1	51	166	164	7	13	49	59	2
16_80650805	A/G	rs13329835	Michailidou, K. et al. (2013)	126	107	22	0	18	20	4	0	196	158	34	0	62	48	13	0
16_87085237	C/A	rs4496150	Michailidou, K et al. (2017)	145	93	17	0	25	14	3	0	235	134	19	0	70	49	4	0
17_29230521	GT/-	rs146699004	Finucane, H. K. et al. (2015)	NA	NA	NA	NA	NA	NA	NA	NA	NA	NA	NA	NA	NA	NA	NA	NA
17_40836389	C/T	rs72826962	Michailidou, K et al. (2017)	247	8	0	0	40	2	0	0	383	5	0	0	119	4	0	0
17_44252468	G/A	rs2532263	Michailidou, K et al. (2017)	NA	NA	NA	NA	NA	NA	NA	NA	NA	NA	NA	NA	NA	NA	NA	NA
17_53209774	A/C	rs2787486	Darabi, H. et al. (2016)	127	105	22	1	17	22	3	0	185	167	35	1	63	50	9	1
17_77781725	A/G	rs745570	Finucane, H. K. et al. (2015)	64	123	68	0	13	16	13	0	99	174	115	0	33	55	35	0
18_24337424	C/G	rs527616	Michailidou, K. et al. (2013)	24	120	111	0	5	18	19	0	43	161	184	0	7	53	63	0
18_24570667	T/G	rs1436904	Michailidou, K. et al. (2013)	93	123	39	0	17	17	8	0	153	183	51	1	50	52	21	0
18_25401205	T/-	rs36194942	Milne et al. (2017)	NA	NA	NA	NA	NA	NA	NA	NA	NA	NA	NA	NA	NA	NA	NA	NA
18_29977689	T/C	rs117618124	Michailidou, K et al. (2017)	243	11	1	0	37	5	0	0	367	20	1	0	113	9	1	0
18_42399590	A/G	rs6507583	Finucane, H. K. et al. (2015)	217	31	7	0	35	6	1	0	323	59	6	0	95	23	5	0
19_11423703	C/A/G	rs322144	Milne et al. (2017)	56	124	75	0	8	25	9	0	105	172	111	0	29	58	36	0
19_13158277	T/C	rs78269692	Michailidou, K et al. (2017)	229	26	0	0	35	6	1	0	343	44	1	0	110	13	0	0
19_13954571	G/A/C/T	rs2594714	Michailidou, K et al. (2017)	144	81	30	0	20	17	5	0	224	131	33	0	56	51	16	0
19_17401404	C/G	rs67397200	Lawrenson, K. et al. (2016)	125	98	32	0	17	19	6	0	197	152	39	0	61	48	14	0
19_18571141	A/G	rs4808801	Michailidou, K. et al. (2013)	97	127	31	0	15	18	9	0	144	184	60	0	38	65	20	0
19_19545696	G/A/C	rs2965183	Michailidou, K et al. (2017)	107	103	45	0	17	19	6	0	161	164	63	0	51	59	13	0
19_30277729	C/T	rs113701136	Milne et al. (2017)	143	89	23	0	20	17	5	0	185	164	39	0	70	41	12	0
19_44286513	A/G	rs3760982	Michailidou, K. et al. (2013)	57	119	79	0	11	21	10	0	77	192	119	0	28	69	26	0
19_46183033	-/T	rs71338792	Michailidou, K et al. (2017)	NA	NA	NA	NA	NA	NA	NA	NA	NA	NA	NA	NA	NA	NA	NA	NA
20_5948227	G/A	rs16991615	Michailidou, K et al. (2017)	223	31	1	0	36	6	0	0	340	46	2	0	113	10	0	0
20_32588095	T/C/G	rs2284378	Siddiq, A. et al. (2012)	28	102	125	0	4	15	23	0	34	159	195	0	12	57	54	0
20_48945911	A/G	rs6122906	Michailidou, K et al. (2017)	150	79	18	8	25	14	3	0	233	128	19	8	75	37	6	5
21_16520832	G/A	rs2823093	Ghoussaini, M. et al. (2012)	133	104	18	0	20	17	5	0	197	161	30	0	61	49	13	0
22_29121087	A/C/G	rs17879961	Michailidou, K. et al. (2013)	255	0	0	0	42	0	0	0	388	0	0	0	123	0	0	0
22_29621477	C/T	rs132390	Michailidou, K. et al. (2013)	1	17	237	0	0	1	41	0	1	37	350	0	0	13	110	0
22_38568833	C/G/T	rs738321	Michailidou, K et al. (2017)	93	125	37	0	16	17	9	0	141	194	53	0	47	56	20	0
22_39359355		chr22:39359355	Long, J. et al. (2013)	NA	NA	NA	NA	NA	NA	NA	NA	NA	NA	NA	NA	NA	NA	NA	NA
22_40876234	T/C	rs6001930	Michailidou, K. et al. (2013)	208	38	6	3	33	9	0	0	307	73	5	3	103	18	0	2
22_42038786	C/T	rs73161324	Michailidou, K et al. (2017)	234	20	1	0	37	5	0	0	345	42	1	0	111	12	0	0
22_46283297	G/A	rs28512361	Michailidou, K et al. (2017)	NA	NA	NA	NA	NA	NA	NA	NA	NA	NA	NA	NA	NA	NA	NA	NA

Supplementary Table S2. Significant veQTL association in breast tissue

				veQTL		eQTL			
rsNumber	snp_id	ensembl_gene_id	symbol	statistic	p value	statistic	p value	FDR	beta
rs11814448	10_22315843_A_C_b37	ENSG00000228549.2		50.49949	3.80E-19	8.070683	2.83E-14	4.58E-08	0.150048
rs11814448	10_22315843_A_C_b37	ENSG00000225972.1	*MTND1P23*	42.17759	1.65E-16	10.23194	8.68E-21	7.54E-14	452.9801
rs11814448	10_22315843_A_C_b37	ENSG00000237973.1	*MTCO1P12*	31.00278	9.38E-13	7.012796	2.12E-11	1.08E-05	178.9091
rs11814448	10_22315843_A_C_b37	ENSG00000259187.1		30.05232	2.01E-12	9.322624	5.86E-18	3.71E-11	0.08945
rs6569648	6_130349119_C_T_b37	ENSG00000198945.3	*L3MBTL3*	29.63327	2.77E-12	-6.82825	6.35E-11	2.59E-05	-0.91417
rs11814448	10_22315843_A_C_b37	ENSG00000233421.3	*LINC01783*	29.59542	2.91E-12	6.813301	6.93E-11	2.81E-05	0.068533
rs4577244	2_29120733_C_T_b37	ENSG00000265550.1		29.42142	3.32E-12	3.669368	0.000296	0.461097	0.020877
rs11814448	10_22315843_A_C_b37	ENSG00000132967.9	*HMGB1P5*	28.98688	4.77E-12	7.113295	1.16E-11	6.41E-06	1.433659
rs11814448	10_22315843_A_C_b37	ENSG00000232177.1	*MTND4P24*	28.17592	9.22E-12	9.014549	5.01E-17	2.72E-10	0.88523
rs2594714	19_13954571_G_A_b37	ENSG00000225972.1	*MTND1P23*	27.61898	1.43E-11	6.848786	5.63E-11	2.37E-05	263.147
rs2290203	15_91512067_G_A_b37	ENSG00000233870.1		26.42601	3.81E-11	4.487135	1.10E-05	0.131247	0.060856
rs3817198	11_1909006_T_C_b37	ENSG00000204936.5	*CD177*	25.95186	5.65E-11	4.950537	1.35E-06	0.040025	0.413441
rs11814448	10_22315843_A_C_b37	ENSG00000270773.1		24.49176	1.93E-10	7.821325	1.42E-13	1.70E-07	0.104624
rs1011970	9_22062134_G_T_b37	ENSG00000137440.3	*FGFBP1*	24.44456	2.00E-10	NA	NA	NA	NA
rs34005590	2_217963060_C_A_b37	ENSG00000167748.6	*KLK1*	42.08075	4.60E-10	6.07779	4.46E-09	0.000647	0.623623
rs1011970	9_22062134_G_T_b37	ENSG00000267409.1		22.98151	6.84E-10	3.925659	0.000112	0.349501	0.022072
rs11075995	16_53855291_A_T_b37	ENSG00000147465.7	*STAR*	22.89332	7.32E-10	-3.16488	0.001741	0.671939	-0.31434
rs13329835	16_80650805_A_G_b37	ENSG00000223715.1	*LINC01208*	22.72902	8.41E-10	4.731946	3.70E-06	0.072436	0.06631
rs2594714	19_13954571_G_A_b37	ENSG00000172799.5	*ZBTB8OSP2*	22.69605	8.65E-10	5.501983	9.19E-08	0.006338	0.090718
rs2594714	19_13954571_G_A_b37	ENSG00000196656.6		22.6475	9.01E-10	5.821312	1.76E-08	0.001849	0.772953
rs11814448	10_22315843_A_C_b37	ENSG00000204666.3		22.62745	9.29E-10	7.458999	1.39E-12	1.05E-06	0.138107
rs11075995	16_53855291_A_T_b37	ENSG00000148795.5	*CYP17A1*	22.58008	9.54E-10	-3.26529	0.001245	0.633863	-0.51502
rs117618124	18_29977689_T_C_b37	ENSG00000256609.1		39.73104	1.29E-09	4.408907	1.54E-05	0.155049	0.361254
rs71557345	6_26680698_G_A_b37	ENSG00000106333.8	*PCOLCE*	39.31444	1.61E-09	3.321224	0.001028	0.612003	22.33045
rs11814448	10_22315843_A_C_b37	ENSG00000237533.1		21.95426	1.64E-09	6.866581	5.07E-11	2.19E-05	0.063607
rs6828523	4_175846426_C_A_b37	ENSG00000236055.1		39.24253	1.66E-09	3.053222	0.002506	0.710434	0.06774
rs11814448	10_22315843_A_C_b37	ENSG00000241717.1	*VWFP1*	21.88377	1.75E-09	7.180953	7.70E-12	4.43E-06	0.100485
rs71557345	6_26680698_G_A_b37	ENSG00000198796.6	*ALPK2*	39.11124	1.76E-09	4.535268	8.89E-06	0.117137	0.2051
rs2594714	19_13954571_G_A_b37	ENSG00000232177.1	*MTND4P24*	21.81229	1.83E-09	6.866379	5.07E-11	2.19E-05	0.565481
rs6815814	4_38816338_A_C_b37	ENSG00000204666.3		21.58118	2.23E-09	5.678708	3.72E-08	0.003214	0.084691
rs45631563	10_123349324_A_T_b37	ENSG00000166923.6	*GREM1*	38.32309	2.42E-09	5.230194	3.55E-07	0.016941	11.50943
rs11814448	10_22315843_A_C_b37	ENSG00000225093.1	*RPL3P7*	21.24029	3.02E-09	6.464321	5.20E-10	0.000133	1.830376
rs4442975	2_217920769_G_T_b37	ENSG00000196355.2		20.88245	4.06E-09	2.886584	0.004231	0.761478	0.223883
rs11075995	16_53855291_A_T_b37	ENSG00000203859.5	*HSD3B2*	20.79352	4.38E-09	-3.12874	0.001961	0.684571	-0.12752
rs3817198	11_1909006_T_C_b37	ENSG00000163221.7	*S100A12*	20.77607	4.45E-09	4.648081	5.40E-06	0.08912	2.190524
rs71557345	6_26680698_G_A_b37	ENSG00000182759.3	*MAFA*	36.76739	4.98E-09	3.900037	0.000123	0.359677	0.041186
rs13365225	8_36858483_A_G_b37	ENSG00000233845.1		20.23064	7.11E-09	2.953457	0.003438	0.741915	0.050853
rs11814448	10_22315843_A_C_b37	ENSG00000172799.5	*ZBTB8OSP2*	20.15062	7.70E-09	7.885762	9.37E-14	1.28E-07	0.155109
rs4849887	2_121245122_T_C_b37	ENSG00000270388.1	*MTCO3P22*	35.66349	8.23E-09	-5.16983	4.76E-07	0.020325	-0.04377
rs117618124	18_29977689_T_C_b37	ENSG00000075886.10	*TUBA3D*	35.35543	9.12E-09	4.940095	1.42E-06	0.041209	0.5659
rs2594714	19_13954571_G_A_b37	ENSG00000228549.2		19.78233	1.05E-08	5.207097	3.97E-07	0.018161	0.081989
rs11814448	10_22315843_A_C_b37	ENSG00000259781.1	*HMGB1P6*	19.72608	1.11E-08	3.282477	0.001174	0.627307	1.012496
rs12479355	2_227226952_A_G_b37	ENSG00000227135.1		19.58359	1.24E-08	3.285911	0.00116	0.625825	0.057177
rs11075995	16_53855291_A_T_b37	ENSG00000160882.7	*CYP11B1*	19.43908	1.41E-08	-2.96472	0.003319	0.738206	-0.27821
rs10816625	9_110837073_A_G_b37	ENSG00000207574.1	*MIR661*	34.39197	1.43E-08	4.298603	2.45E-05	0.192719	0.037329
rs10069690	5_1279790_C_T_b37	ENSG00000237973.1	*MTCO1P12*	19.38508	1.48E-08	5.08253	7.25E-07	0.026904	118.2104
rs2594714	19_13954571_G_A_b37	ENSG00000132967.9	*HMGB1P5*	19.34181	1.53E-08	4.745495	3.48E-06	0.069968	0.797869
rs4808801	19_18571141_A_G_b37	ENSG00000206192.7	*ANKRD20A9P*	18.97125	2.11E-08	4.685904	4.56E-06	0.081194	0.038842
rs13365225	8_36858483_A_G_b37	ENSG00000183628.8	*DGCR6*	18.94781	2.16E-08	3.472484	0.000606	0.548699	1.182263
rs11814448	10_22315843_A_C_b37	ENSG00000198868.3	*MTND4LP30*	18.9325	2.21E-08	3.780043	0.000196	0.412229	0.564712
rs2594714	19_13954571_G_A_b37	ENSG00000270773.1		18.88106	2.29E-08	4.712511	4.04E-06	0.076137	0.053525
rs1011970	9_22062134_G_T_b37	ENSG00000259342.1		18.81922	2.42E-08	3.478033	0.000594	0.546313	0.57306
rs2594714	19_13954571_G_A_b37	ENSG00000250133.2	*HOXC-AS2*	18.78833	2.48E-08	4.509371	9.95E-06	0.124601	0.075172
rs2594714	19_13954571_G_A_b37	ENSG00000233421.3	*LINC01783*	18.76005	2.54E-08	4.54145	8.65E-06	0.115926	0.037975
rs11814448	10_22315843_A_C_b37	ENSG00000232573.1	*RPL3P4*	18.68325	2.74E-08	6.07401	4.55E-09	0.000659	22.00195
rs13329835	16_80650805_A_G_b37	ENSG00000230202.1		18.65382	2.79E-08	5.194654	4.22E-07	0.018834	9.754592
rs13329835	16_80650805_A_G_b37	ENSG00000254052.1	*IGHVIII-67-4*	18.64176	2.82E-08	5.072465	7.60E-07	0.027849	0.023768
rs71557345	6_26680698_G_A_b37	ENSG00000269657.1		32.79102	2.98E-08	4.355079	1.93E-05	0.173373	0.284512
rs6122906	20_48945911_A_G_b37	ENSG00000213959.2		18.61534	2.99E-08	NA	NA	NA	NA
rs6596100	5_132407058_C_T_b37	ENSG00000222040.3		18.55912	3.03E-08	NA	NA	NA	NA
rs7297051	12_28174817_C_T_b37	ENSG00000253631.1	*IGLV7-35*	18.41294	3.44E-08	3.424007	0.00072	0.570933	0.035082
rs132390	22_29621477_C_T_b37	ENSG00000273179.1		32.33548	3.58E-08	-4.16862	4.21E-05	0.242707	-5.71457
rs9348512	6_10456706_C_A_b37	ENSG00000242163.1		18.30041	3.79E-08	NA	NA	NA	NA
rs6596100	5_132407058_C_T_b37	ENSG00000199480.1	*RNA5SP389*	18.23765	4.01E-08	3.365525	0.000883	0.595057	0.019393
rs71557345	6_26680698_G_A_b37	ENSG00000164692.13	*COL1A2*	32.07318	4.13E-08	4.01269	7.91E-05	0.311115	146.1247
rs13329835	16_80650805_A_G_b37	ENSG00000196656.6		18.1913	4.17E-08	4.847704	2.18E-06	0.053351	0.707769
rs117618124	18_29977689_T_C_b37	ENSG00000130202.5	*NECTIN2*	31.87789	4.41E-08	3.234294	0.001382	0.646587	10.24472

rs71557345	6_26680698_G_A_b37	ENSG00000174429.3	*ABRA*	31.87674	4.52E-08	3.951197	0.000101	0.337694	0.140718
rs71557345	6_26680698_G_A_b37	ENSG00000104369.4	*JPH1*	31.78829	4.70E-08	3.887881	0.000129	0.365092	0.571313
rs117618124	18_29977689_T_C_b37	ENSG00000152086.7	*TUBA3E*	31.73946	4.70E-08	4.929476	1.49E-06	0.042312	0.596702

Supplementary Table S3. Significant veQTL association in lung tissue

				veQTL		eQTL			
rsNumber	snp_id	ensembl_gene_id	symbol	statistic	p.value	statistic	p value	FDR	beta
rs11814448	10_22315843_A_C_b37	ENSG00000241717.1	VWFP1	48.07036	2.48E-19	9.344079	7.56E-19	2.21E-12	0.154989
rs11814448	10_22315843_A_C_b37	ENSG00000225972.1	MTND1P23	47.39222	4.26E-19	9.582612	1.19E-19	3.96E-13	243.4222
rs6815814	4_38816338_A_C_b37	ENSG00000225972.1	MTND1P23	43.48135	9.42E-18	7.899303	2.95E-14	2.75E-08	150.4213
rs11814448	10_22315843_A_C_b37	ENSG00000228549.2		38.76695	4.81E-16	6.886425	2.33E-11	6.84E-06	0.124904
rs11814448	10_22315843_A_C_b37	ENSG00000178084.1	HTR3C	38.18955	7.77E-16	8.436285	6.65E-16	8.98E-10	3.524893
rs11814448	10_22315843_A_C_b37	ENSG00000232573.1	RPL3P4	37.90991	9.81E-16	7.934273	2.32E-14	2.18E-08	13.47634
rs11814448	10_22315843_A_C_b37	ENSG00000198794.7	SCAMP5	36.07956	4.54E-15	7.71724	1.03E-13	8.10E-08	0.721697
rs6815814	4_38816338_A_C_b37	ENSG00000232177.1	MTND4P24	33.87481	2.81E-14	6.679682	8.38E-11	1.96E-05	0.539107
rs11814448	10_22315843_A_C_b37	ENSG00000237973.1	MTCO1P12	33.89004	2.88E-14	8.109757	6.81E-15	7.33E-09	202.1534
rs11814448	10_22315843_A_C_b37	ENSG00000225093.1	RPL3P7	33.89003	2.88E-14	7.441784	6.52E-13	3.88E-07	0.989202
rs2594714	19_13954571_G_A_b37	ENSG00000241717.1	VWFP1	31.12784	2.95E-13	7.634281	1.80E-13	1.30E-07	0.103079
rs11780156	8_129194641_C_T_b37	ENSG00000210191.1	MT-TL2	30.29678	6.03E-13	4.033029	6.64E-05	0.305223	3.032586
rs2594714	19_13954571_G_A_b37	ENSG00000225972.1	MTND1P23	29.71902	9.94E-13	7.537735	3.44E-13	2.26E-07	156.8679
rs13267382	8_117209548_A_G_b37	ENSG00000241717.1	VWFP1	29.02322	1.82E-12	-6.429	3.80E-10	6.59E-05	-0.07719
rs11814448	10_22315843_A_C_b37	ENSG00000259187.1		27.47792	7.18E-12	7.541799	3.35E-13	2.23E-07	0.056837
rs13267382	8_117209548_A_G_b37	ENSG00000225972.1	MTND1P23	27.30935	8.11E-12	-6.45564	3.24E-10	5.85E-05	-119.218
rs6815814	4_38816338_A_C_b37	ENSG00000259781.1	HMGB1P6	26.77915	1.29E-11	NA	NA	NA	NA
rs10069690	5_1279790_C_T_b37	ENSG00000237973.1	MTCO1P12	26.63318	1.47E-11	5.61167	3.83E-08	0.002647	117.4359
rs11814448	10_22315843_A_C_b37	ENSG00000237533.1		26.4965	1.70E-11	7.281702	1.87E-12	8.61E-07	0.040969
rs11814448	10_22315843_A_C_b37	ENSG00000132967.9	HMGB1P5	26.33039	1.96E-11	5.361441	1.42E-07	0.007096	0.801539
rs11117758	1_217220574_G_A_b37	ENSG00000225840.1		25.95848	2.66E-11	4.06847	5.74E-05	0.2879	0.055603
rs11814448	10_22315843_A_C_b37	ENSG00000196656.6		25.71532	3.37E-11	8.74484	6.97E-17	1.28E-10	0.786223
rs13294895	9_110837176_C_T_b37	ENSG00000152592.9	DMP1	25.38528	4.42E-11	3.526869	0.000471	0.534611	0.017648
rs11814448	10_22315843_A_C_b37	ENSG00000172799.5	ZBTB8OSP2	25.1106	5.74E-11	8.124525	6.14E-15	6.69E-09	0.150174
rs11814448	10_22315843_A_C_b37	ENSG00000260581.1		25.01726	6.24E-11	6.518125	2.23E-10	4.28E-05	0.124368
rs11814448	10_22315843_A_C_b37	ENSG00000206561.8	COLQ	25.00467	6.30E-11	6.395938	4.62E-10	7.76E-05	1.166548
rs1011970	9_22062134_G_T_b37	ENSG00000116031.7	CD207	24.65089	8.44E-11	3.396033	0.000755	0.587538	0.753267
rs11552449	1_114448389_C_T_b37	ENSG00000128510.6	CPA4	24.39369	1.06E-10	3.976074	8.36E-05	0.331668	0.858504
rs13329835	16_80650805_A_G_b37	ENSG00000230202.1		24.36172	1.09E-10	3.786316	0.000177	0.419547	3.155778
rs2594714	19_13954571_G_A_b37	ENSG00000178084.1	HTR3C	24.35691	1.10E-10	5.110158	5.07E-07	0.018322	1.773593
rs11552449	1_114448389_C_T_b37	ENSG00000154736.5	ADAMTS5	24.19458	1.27E-10	3.293957	0.001079	0.627726	0.242725
rs13294895	9_110837176_C_T_b37	ENSG00000222078.1	RN7SKP110	24.16669	1.30E-10	3.676324	0.00027	0.470237	0.039695
rs13267382	8_117209548_A_G_b37	ENSG00000226278.1	PSPHP1	23.6323	2.09E-10	-5.95877	5.73E-09	0.000585	-0.49108
rs11814448	10_22315843_A_C_b37	ENSG00000227939.1	RPL3P2	23.52492	2.34E-10	5.92392	6.96E-09	0.000684	0.327917
rs17156577	7_28356889_T_C_b37	ENSG00000224690.2	UBE2D3P3	23.25173	2.93E-10	3.244677	0.001278	0.646623	0.048914
rs6815814	4_38816338_A_C_b37	ENSG00000241717.1	VWFP1	23.14449	3.22E-10	6.301117	8.07E-10	0.00012	0.080028
rs11780156	8_129194641_C_T_b37	ENSG00000207870.1	MIR221	22.95775	3.81E-10	4.172661	3.72E-05	0.241997	1.788219
rs13267382	8_117209548_A_G_b37	ENSG00000172799.5	ZBTB8OSP2	22.82846	4.27E-10	-5.27725	2.19E-07	0.009857	-0.07011
rs11814448	10_22315843_A_C_b37	ENSG00000230202.1		22.22426	7.47E-10	5.694664	2.45E-08	0.001871	5.87547
rs999737	14_69034682_C_T_b37	ENSG00000139648.6	KRT71	22.0635	8.48E-10	3.646598	0.000302	0.484033	0.016778
rs13294895	9_110837176_C_T_b37	ENSG00000230491.1		21.97466	9.21E-10	4.32884	1.91E-05	0.180035	0.100809
rs7297051	12_28174817_C_T_b37	ENSG00000229647.1	MYOSLID	21.69091	1.19E-09	2.752418	0.006195	0.793258	0.043941
rs2236007	14_37132769_G_A_b37	ENSG00000222100.1	<NA>	21.59854	1.29E-09	3.688976	0.000257	0.464136	0.016441
rs11814448	10_22315843_A_C_b37	ENSG00000259781.1	HMGB1P6	21.26141	1.77E-09	NA	NA	NA	NA
rs11780156	8_129194641_C_T_b37	ENSG00000202382.1		21.13495	1.95E-09	NA	NA	NA	NA
rs10069690	5_1279790_C_T_b37	ENSG00000225972.1	MTND1P23	21.06764	2.08E-09	5.348539	1.52E-07	0.007451	117.7144
rs17156577	7_28356889_T_C_b37	ENSG00000196656.6		21.04587	2.12E-09	4.93877	1.17E-06	0.0333	0.464248
rs6597981	11_803017_A_G_b37	ENSG00000177236.3		20.96078	2.29E-09	11.10352	4.79E-25	5.97E-18	0.983789
rs2594714	19_13954571_G_A_b37	ENSG00000254052.1	IGHVIII-67-4	20.95391	2.30E-09	5.795953	1.41E-08	0.001221	0.057248
rs74911261	11_108357137_G_A_b37	ENSG00000206727.1	SNORD116-9	37.40349	2.36E-09	6.085906	2.79E-09	0.000329	0.054712
rs17156577	7_28356889_T_C_b37	ENSG00000223070.1		20.90533	2.40E-09	3.975564	8.38E-05	0.331977	0.012109
rs11814448	10_22315843_A_C_b37	ENSG00000261701.2	HPR	20.87609	2.51E-09	4.851883	1.78E-06	0.044635	1.314762
rs11814448	10_22315843_A_C_b37	ENSG00000254052.1	IGHVIII-67-4	20.8191	2.64E-09	5.40202	1.15E-07	0.006062	0.068013
rs11780156	8_129194641_C_T_b37	ENSG00000270071.1		20.69178	2.91E-09	3.814128	0.000159	0.40623	0.036058
rs6815814	4_38816338_A_C_b37	ENSG00000132967.9	HMGB1P5	20.67753	2.95E-09	NA	NA	NA	NA
rs4784227	16_52599188_C_T_b37	ENSG00000225217.1	HSPA7	20.57101	3.25E-09	3.447975	0.000627	0.56683	6.96896
rs2594714	19_13954571_G_A_b37	ENSG00000259781.1	HMGB1P6	20.39878	3.80E-09	NA	NA	NA	NA
rs17156577	7_28356889_T_C_b37	ENSG00000228549.2		20.35245	3.96E-09	4.189884	3.46E-05	0.234735	0.077568
rs10941679	5_44706498_A_G_b37	ENSG00000111536.4	IL26	20.26428	4.32E-09	3.862668	0.000131	0.384817	0.033917
rs11814448	10_22315843_A_C_b37	ENSG00000272824.1		20.16611	4.76E-09	5.769261	1.64E-08	0.001376	0.225647
rs11814448	10_22315843_A_C_b37	ENSG00000233368.2		20.02066	5.43E-09	5.916629	7.25E-09	0.000709	0.080655
rs11780156	8_129194641_C_T_b37	ENSG00000113327.10	GABRG2	19.90954	5.91E-09	3.381338	0.000795	0.593875	0.016549
rs1011970	9_22062134_G_T_b37	ENSG00000207442.1	SNORD116-6	19.88538	6.05E-09	4.559791	6.88E-06	0.104327	0.016294
rs4784227	16_52599188_C_T_b37	ENSG00000152049.5	KCNE4	19.77723	6.67E-09	3.134752	0.001852	0.684777	0.62801
rs2236007	14_37132769_G_A_b37	ENSG00000260507.1		19.70397	7.13E-09	3.958326	8.98E-05	0.340188	0.038948
rs7707921	5_81538046_T_A_b37	ENSG00000211782.2	TRAV8-1	19.58772	7.94E-09	-3.69481	0.000252	0.461519	-0.0766
rs28539243	16_54682064_G_A_b37	ENSG00000226278.1	PSPHP1	19.55908	8.13E-09	4.788162	2.40E-06	0.054583	0.414862

rs2588809	14_68660428_T_C_b37	ENSG00000265676.1		19.49504	8.65E-09	-3.13069	0.001877	0.686339	-0.01432
rs17156577	7_28356889_T_C_b37	ENSG00000167619.7	TMEM145	19.42529	9.18E-09	3.136336	0.001842	0.684452	0.121084
rs13329835	16_80650805_A_G_b37	ENSG00000230869.1	AGAP10P	19.40416	9.36E-09	4.207555	3.21E-05	0.226955	0.043739
rs17156577	7_28356889_T_C_b37	ENSG00000254052.1	IGHVIII-67-4	19.38882	9.49E-09	3.050309	0.002444	0.712487	0.038764
rs72755295	1_242034263_A_G_b37	ENSG00000201635.1		33.88949	1.23E-08	6.09942	2.59E-09	0.00031	0.066044
rs74911261	11_108357137_G_A_b37	ENSG00000130383.6	FUT5	33.75655	1.31E-08	5.754997	1.77E-08	0.001465	0.072941
rs117618124	18_29977689_T_C_b37	ENSG00000162493.12	PDPN	33.71822	1.33E-08	5.518265	6.29E-08	0.003842	10.30805
rs7707921	5_81538046_T_A_b37	ENSG00000238612.1		18.9309	1.44E-08	-3.60004	0.00036	0.504298	-0.02177
rs6815814	4_38816338_A_C_b37	ENSG00000237973.1	MTCO1P12	18.89535	1.49E-08	5.632257	3.43E-08	0.002427	106.0257
rs6815814	4_38816338_A_C_b37	ENSG00000204894.4		18.87397	1.52E-08	5.532059	5.85E-08	0.003641	0.138007
rs7297051	12_28174817_C_T_b37	ENSG00000264201.1	MIR4701	18.84331	1.56E-08	3.549299	0.000434	0.525818	0.017805
rs72755295	1_242034263_A_G_b37	ENSG00000199291.1		33.33257	1.60E-08	5.786668	1.49E-08	0.001274	0.083252
rs17156577	7_28356889_T_C_b37	ENSG00000178084.1	HTR3C	18.73725	1.72E-08	5.282957	2.13E-07	0.009619	2.28482
rs941764	14_91841069_A_G_b37	ENSG00000225972.1	MTND1P23	18.62881	1.89E-08	4.724908	3.23E-06	0.065884	95.7224
rs13267382	8_117209548_A_G_b37	ENSG00000237533.1		18.62085	1.91E-08	-4.77063	2.61E-06	0.057564	-0.01913
rs11814448	10_22315843_A_C_b37	ENSG00000238137.2	ARPC3P2	18.53248	2.09E-08	5.627591	3.52E-08	0.002477	0.021362
rs10069690	5_1279790_C_T_b37	ENSG00000237533.1		18.5111	2.11E-08	5.381569	1.28E-07	0.006545	0.025134
rs11814448	10_22315843_A_C_b37	ENSG00000232177.1	MTND4P24	18.46772	2.22E-08	6.765372	4.95E-11	1.28E-05	0.750912
rs11814448	10_22315843_A_C_b37	ENSG00000241278.1	ENPP7P4	18.45918	2.24E-08	4.033609	6.62E-05	0.304964	0.095627
rs117618124	18_29977689_T_C_b37	ENSG00000166923.6	GREM1	32.58509	2.28E-08	5.388265	1.24E-07	0.006385	10.46782
rs10069690	5_1279790_C_T_b37	ENSG00000238533.1		18.35506	2.43E-08	4.078948	5.50E-05	0.283452	0.020669
rs11780156	8_129194641_C_T_b37	ENSG00000152954.7	NRSN1	18.31527	2.52E-08	3.395643	0.000756	0.587654	0.013267
rs999737	14_69034682_C_T_b37	ENSG00000212899.1	KRTAP3-3	18.24272	2.69E-08	3.411733	0.000714	0.581433	0.024629
rs12422552	12_14413931_G_C_b37	ENSG00000202141.1		18.22187	2.75E-08	3.629741	0.000322	0.491845	0.027855
rs71557345	6_26680698_G_A_b37	ENSG00000269468.1		32.05295	2.98E-08	5.326647	1.70E-07	0.008126	0.062896
rs6815814	4_38816338_A_C_b37	ENSG00000226278.1	PSPHP1	18.08665	3.11E-08	6.522907	2.17E-10	4.18E-05	0.562894
rs11780156	8_129194641_C_T_b37	ENSG00000161082.8	CELF5	18.02301	3.29E-08	3.114608	0.001979	0.691603	0.013582
rs11780156	8_129194641_C_T_b37	ENSG00000202190.1		18.01421	3.32E-08	2.763211	0.005998	0.790914	0.013526
rs11814448	10_22315843_A_C_b37	ENSG00000230869.1	AGAP10P	17.94178	3.59E-08	5.731487	2.01E-08	0.001624	0.074027
rs11780156	8_129194641_C_T_b37	ENSG00000167654.13	ATCAY	17.91211	3.65E-08	3.202418	0.001476	0.662114	0.018803
rs2594714	19_13954571_G_A_b37	ENSG00000232573.1	RPL3P4	17.87605	3.77E-08	4.738196	3.04E-06	0.0634	6.654305
rs999737	14_69034682_C_T_b37	ENSG00000221880.2	KRTAP1-3	17.86728	3.80E-08	3.056282	0.002397	0.710435	0.017833
rs117618124	18_29977689_T_C_b37	ENSG00000047457.9	CP	31.36431	4.08E-08	3.925196	0.000103	0.355944	4.415866
rs7707921	5_81538046_T_A_b37	ENSG00000267406.1		17.75664	4.21E-08	-3.16442	0.001677	0.675344	-0.01309
rs999737	14_69034682_C_T_b37	ENSG00000131737.5	KRT34	17.72364	4.33E-08	2.88957	0.004075	0.759159	0.014288
rs11814448	10_22315843_A_C_b37	ENSG00000243289.1	AGAP13P	17.732	4.35E-08	6.085754	2.80E-09	0.000329	0.074291
rs17529111	6_82128386_T_C_b37	ENSG00000121742.11	GJB6	17.71702	4.40E-08	NA	NA	NA	NA
rs11780156	8_129194641_C_T_b37	ENSG00000223027.1		17.68804	4.48E-08	2.690943	0.007435	0.807178	0.020651
rs11780156	8_129194641_C_T_b37	ENSG00000222691.1	RNU6-733P	17.65281	4.62E-08	3.551042	0.000431	0.525085	0.043929
rs7707921	5_81538046_T_A_b37	ENSG00000259389.2	H3F3AP1	17.63997	4.69E-08	-5.03006	7.53E-07	0.024408	-0.01712
rs11814448	10_22315843_A_C_b37	ENSG00000253239.1	IGLVI-70	17.6256	4.79E-08	4.068065	5.75E-05	0.288092	1.24087
rs11814448	10_22315843_A_C_b37	ENSG00000253228.1	NRBF2P4	17.59504	4.93E-08	6.186086	1.57E-09	0.00021	0.019089

Supplementary Table S4. Significant veQTL association in ovarian tissue

				veQTL		eQTL			
rsNumber	snp_id	ensembl_gene_id	symbol	statistic	p.value	statistic	pvalue	FDR	beta
rs11814448	10_22315843_A_C_b37	ENSG00000232177.1	*MTND4P24*	65.61558	7.44E-13	9.06132	2.77E-15	9.26E-08	0.881866
rs12710696	2_19320803_T_C_b37	ENSG00000237073.1		30.20368	2.37E-11	-4.23316	4.51E-05	0.44152	-0.02959
rs1830298	2_202181247_C_T_b37	ENSG00000242371.1	*IGKV1-39*	25.98798	4.20E-10	-4.91565	2.81E-06	0.170692	-1.84936
rs13294895	9_110837176_C_T_b37	ENSG00000249650.1		42.92236	1.46E-09	3.810349	0.00022	0.609343	0.047022
rs1830298	2_202181247_C_T_b37	ENSG00000137078.4	*SIT1*	23.44881	2.53E-09	-4.13232	6.65E-05	0.483321	-0.08871
rs11814448	10_22315843_A_C_b37	ENSG00000259781.1	*HMGB1P6*	38.7783	8.51E-09	NA	NA	NA	NA
rs11814448	10_22315843_A_C_b37	ENSG00000272824.1		38.33455	1.01E-08	NA	NA	NA	NA
rs11814448	10_22315843_A_C_b37	ENSG00000099365.5	*STX1B*	36.86581	1.77E-08	4.621709	9.59E-06	0.277395	1.075658
rs6597981	11_803017_A_G_b37	ENSG00000177236.3	*NA*	19.58818	4.35E-08	7.984752	9.19E-13	2.83E-06	1.537733

Supplementary Table S5. Traits associated with breast cancer risk variants identified through www.gwascentral.org

GWAS central Study Identifier	Study name	rsNumber	Chr	Start	p value	-log.p	Annotation name
HGVST1616	GWAS of Breast cancer	rs616488	1	10566215	2.00E-10	9.699	Breast Neoplasms
HGVST1622	GWAS of Breast cancer	rs616488	1	10566215	1.00E-08	8	Breast Neoplasms
HGVST2054	GWAS of Breast cancer	rs616488	1	10566215	5.00E-20	19.301	Breast Neoplasms
HGVST2403	GWAS of Breast cancer	rs616488	1	10566215	4.00E-18	17.398	Breast Neoplasms
HGVST2746	GWAS of Breast cancer	rs616488	1	10566215	4.00E-11	10.398	Breast Neoplasms
HGVST2054	GWAS of Breast cancer	rs2992756	1	18807339	2.00E-15	14.699	Breast Neoplasms
HGVST2054	GWAS of Breast cancer	rs4233486	1	41380440	9.00E-09	8.046	Breast Neoplasms
HGVST2054	GWAS of Breast cancer	rs79724016	1	42137311	4.00E-08	7.398	Breast Neoplasms
HGVST2054	GWAS of Breast cancer	rs1707302	1	46600917	3.00E-08	7.523	Breast Neoplasms
HGVST2054	GWAS of Breast cancer	rs140850326	1	50846033	4.00E-08	7.398	Breast Neoplasms
HGVST2054	GWAS of Breast cancer	rs17426269	1	88156923	2.00E-08	7.699	Breast Neoplasms
HGVST1616	GWAS of Breast cancer	rs11552449	1	1.14E+08	2.00E-08	7.699	Breast Neoplasms
HGVST2054	GWAS of Breast cancer	rs11552449	1	1.14E+08	5.00E-11	10.301	Breast Neoplasms
HGVST2746	GWAS of Breast cancer	rs11552449	1	1.14E+08	3.00E-09	8.523	Breast Neoplasms
HGVST3483	GWAS of Longevity	rs11552449	1	1.14E+08	6.00E-06	5.222	Longevity
HGVST2054	GWAS of Breast cancer	rs7529522	1	1.18E+08	2.00E-10	9.699	Breast Neoplasms
HGVST155	GWAS of breast cancer	rs11249433	1	1.21E+08	7.00E-10	9.15490196	Breast Neoplasms
HGVST1616	GWAS of Breast cancer	rs11249433	1	1.21E+08	2.00E-26	25.699	Breast Neoplasms
HGVST2054	GWAS of Breast cancer	rs11249433	1	1.21E+08	2.00E-52	51.699	Breast Neoplasms
HGVST2746	GWAS of Breast cancer	rs11249433	1	1.21E+08	3.00E-27	26.523	Breast Neoplasms
HGVST2054	GWAS of Breast cancer	rs12405132	1	1.46E+08	6.00E-10	9.222	Breast Neoplasms
HGVST2746	GWAS of Breast cancer	rs12405132	1	1.46E+08	8.00E-09	8.097	Breast Neoplasms
HGVST2054	GWAS of Breast cancer	rs12048493	1	1.5E+08	9.00E-14	13.046	Breast Neoplasms
HGVST2746	GWAS of Breast cancer	rs12048493	1	1.5E+08	1.00E-09	9	Breast Neoplasms
HGVST3399	GWAS of Heel bone mineral density	rs12048493	1	1.5E+08	1.00E-17	17	Bone Density
HGVST2054	GWAS of Breast cancer	rs4971059	1	1.55E+08	5.00E-11	10.301	Breast Neoplasms
HGVST2054	GWAS of Breast cancer	rs35383942	1	2.01E+08	4.00E-13	12.398	Breast Neoplasms
HGVST3598	GWAS of Male-pattern baldness	rs35383942	1	2.01E+08	2.00E-15	14.699	Alopecia
HGVST1622	GWAS of Breast cancer	rs6678914	1	2.02E+08	1.00E-08	8	Breast Neoplasms
HGVST2403	GWAS of Breast cancer	rs6678914	1	2.02E+08	3.00E-12	11.523	Breast Neoplasms
HGVST2447	GWAS of Breast cancer	rs4951011	1	2.04E+08	9.00E-09	8.046	Breast Neoplasms
HGVST1622	GWAS of Breast cancer	rs4245739	1	2.05E+08	2.00E-12	11.699	Breast Neoplasms
HGVST1690	GWAS of Prostate cancer	rs4245739	1	2.05E+08	2.00E-11	10.699	Prostatic Neoplasms
HGVST2285	GWAS of Prostate cancer	rs4245739	1	2.05E+08	3.00E-24	23.523	Prostatic Neoplasms
HGVST2318	GWAS of Breast cancer	rs4245739	1	2.05E+08	4.00E-06	5.398	Triple Negative Breast Neoplasms
HGVST2403	GWAS of Breast cancer	rs4245739	1	2.05E+08	3.00E-23	22.523	Breast Neoplasms
HGVST3137	GWAS of Breast cancer	rs4245739	1	2.05E+08	6.00E-15	14.222	Breast Neoplasms
HGVST3137	GWAS of Breast cancer	rs4245739	1	2.05E+08	8.00E-18	17.097	Breast Neoplasms
HGVST3563	GWAS of Smoking behaviour	rs4245739	1	2.05E+08	2.00E-09	8.699	Blood Pressure
HGVST3775	GWAS of Blood pressure	rs4245739	1	2.05E+08	1.00E-06	6	Blood Pressure
HGVST3775	GWAS of Blood pressure	rs4245739	1	2.05E+08	5.00E-09	8.301	Blood Pressure
HGVST2054	GWAS of Breast cancer	rs11117758	1	2.17E+08	4.00E-09	8.398	Breast Neoplasms
HGVST2054	GWAS of Breast cancer	rs72755295	1	2.42E+08	2.00E-14	13.699	Breast Neoplasms
HGVST2746	GWAS of Breast cancer	rs72755295	1	2.42E+08	2.00E-08	7.699	Breast Neoplasms
HGVST3468	GWAS of Various traits	rs72755295	1	2.42E+08	5.00E-13	12.301	Erythrocyte Indices
HGVST3468	GWAS of Various traits	rs72755295	1	2.42E+08	2.00E-10	9.699	Eosinophils
HGVST2054	GWAS of Breast cancer	rs113577745	2	10135681	4.00E-10	9.398	Breast Neoplasms
HGVST1622	GWAS of Breast cancer	rs12710696	2	19320803	5.00E-08	7.301	Breast Neoplasms
HGVST2054	GWAS of Breast cancer	rs12710696	2	19320803	1.00E-08	8	Breast Neoplasms
HGVST2403	GWAS of Breast cancer	rs12710696	2	19320803	7.00E-08	7.155	Breast Neoplasms
HGVST2746	GWAS of Breast cancer	rs12710696	2	19320803	4.00E-07	6.398	Breast Neoplasms
HGVST3137	GWAS of Breast cancer	rs12710696	2	19320803	2.00E-08	7.699	Breast Neoplasms
HGVST3137	GWAS of Breast cancer	rs12710696	2	19320803	2.00E-06	5.699	Breast Neoplasms
HGVST2403	GWAS of Breast cancer	rs200648189	2	24739694	3.00E-07	6.523	Breast Neoplasms
HGVST2403	GWAS of Breast cancer	rs200648189	2	24739694	1.00E-08	8	Breast Neoplasms
HGVST2054	GWAS of Breast cancer	rs6725517	2	25129473	3.00E-12	11.523	Breast Neoplasms
HGVST2403	GWAS of Breast cancer	rs4577244	2	29120733	2.00E-09	8.699	Breast Neoplasms
HGVST2054	GWAS of Breast cancer	rs71801447	2	1.12E+08	4.00E-08	7.398	Breast Neoplasms
HGVST1353	GWAS of Breast size	rs4849887	2	1.21E+08	3.00E-11	10.523	Breast Neoplasms
HGVST1353	GWAS of Breast size	rs4849887	2	1.21E+08	3.00E-11	10.523	Breast
HGVST1616	GWAS of Breast cancer	rs4849887	2	1.21E+08	4.00E-11	10.398	Breast Neoplasms
HGVST2054	GWAS of Breast cancer	rs4849887	2	1.21E+08	7.00E-20	19.155	Breast Neoplasms
HGVST2403	GWAS of Breast cancer	rs4849887	2	1.21E+08	4.00E-09	8.398	Breast Neoplasms
HGVST2746	GWAS of Breast cancer	rs4849887	2	1.21E+08	2.00E-11	10.699	Breast Neoplasms
HGVST1616	GWAS of Breast cancer	rs2016394	2	1.73E+08	1.00E-08	8	Breast Neoplasms
HGVST2054	GWAS of Breast cancer	rs2016394	2	1.73E+08	6.00E-12	11.222	Breast Neoplasms
HGVST2746	GWAS of Breast cancer	rs2016394	2	1.73E+08	8.00E-06	5.097	Breast Neoplasms
HGVST1616	GWAS of Breast cancer	rs1550623	2	1.74E+08	3.00E-08	7.523	Breast Neoplasms
HGVST2054	GWAS of Breast cancer	rs1550623	2	1.74E+08	5.00E-10	9.301	Breast Neoplasms
HGVST2746	GWAS of Breast cancer	rs1550623	2	1.74E+08	1.00E-06	6	Breast Neoplasms
HGVST2054	GWAS of Breast cancer	rs1830298	2	2.02E+08	2.00E-16	15.699	Breast Neoplasms
HGVST2403	GWAS of Breast cancer	rs1830298	2	2.02E+08	7.00E-07	6.155	Breast Neoplasms
HGVST2054	GWAS of Breast cancer	rs4442975	2	2.18E+08	1.00E-95	95	Breast Neoplasms
HGVST2403	GWAS of Breast cancer	rs4442975	2	2.18E+08	3.00E-06	5.523	Breast Neoplasms
HGVST2054	GWAS of Breast cancer	rs34005590	2	2.18E+08	3.00E-41	40.523	Breast Neoplasms
HGVST1616	GWAS of Breast cancer	rs16857609	2	2.18E+08	1.00E-15	15	Breast Neoplasms
HGVST2054	GWAS of Breast cancer	rs16857609	2	2.18E+08	2.00E-25	24.699	Breast Neoplasms
HGVST2403	GWAS of Breast cancer	rs16857609	2	2.18E+08	2.00E-08	7.699	Breast Neoplasms
HGVST2746	GWAS of Breast cancer	rs16857609	2	2.18E+08	1.00E-17	17	Breast Neoplasms
HGVST3137	GWAS of Breast cancer	rs16857609	2	2.18E+08	7.00E-06	5.155	Breast Neoplasms
HGVST2054	GWAS of Breast cancer	rs12479355	2	2.27E+08	2.00E-08	7.699	Breast Neoplasms
HGVST1616	GWAS of Breast cancer	rs6762644	3	4742276	2.00E-12	11.699	Breast Neoplasms
HGVST2054	GWAS of Breast cancer	rs6762644	3	4742276	4.00E-18	17.398	Breast Neoplasms
HGVST2746	GWAS of Breast cancer	rs6762644	3	4742276	9.00E-12	11.046	Breast Neoplasms
HGVST532	GWAS of breast cancer	rs4973768	3	27416013	6.00E-07	6.22184875	Breast Neoplasms
HGVST718	GWAS of breast cancer	rs4973768	3	27416013	2.00E-08	7.698970004	Breast Neoplasms
HGVST1616	GWAS of Breast cancer	rs4973768	3	27416013	2.00E-30	29.699	Breast Neoplasms
HGVST2054	GWAS of Breast cancer	rs4973768	3	27416013	5.00E-57	56.301	Breast Neoplasms
HGVST2096	GWAS of Breast cancer	rs4973768	3	27416013	4.00E-23	22.398	Breast Neoplasms
HGVST2746	GWAS of Breast cancer	rs4973768	3	27416013	3.00E-30	29.523	Breast Neoplasms
HGVST1616	GWAS of Breast cancer	rs12493607	3	30682939	2.00E-08	7.699	Breast Neoplasms
HGVST2054	GWAS of Breast cancer	rs12493607	3	30682939	7.00E-14	13.155	Breast Neoplasms
HGVST2746	GWAS of Breast cancer	rs12493607	3	30682939	1.00E-08	8	Breast Neoplasms
HGVST2054	GWAS of Breast cancer	rs6796502	3	46866866	6.00E-15	14.222	Breast Neoplasms
HGVST2746	GWAS of Breast cancer	rs6796502	3	46866866	2.00E-08	7.699	Breast Neoplasms
HGVST2054	GWAS of Breast cancer	rs1053338	3	63967900	5.00E-11	10.301	Breast Neoplasms
HGVST2746	GWAS of Breast cancer	rs1053338	3	63967900	9.00E-09	8.046	Breast Neoplasms
HGVST2054	GWAS of Breast cancer	rs6805189	3	71532113	5.00E-08	7.301	Breast Neoplasms
HGVST2054	GWAS of Breast cancer	rs13066793	3	87037543	1.00E-09	9	Breast Neoplasms
HGVST2054	GWAS of Breast cancer	rs9833888	3	99723580	5.00E-10	9.301	Breast Neoplasms
HGVST2054	GWAS of Breast cancer	rs34207738	3	1.41E+08	3.00E-15	14.523	Breast Neoplasms

HGVST2054	GWAS of Breast cancer	rs58058861	3	1.72E+08	2.00E-10	9.699	Breast Neoplasms
HGVST2054	GWAS of Breast cancer	rs6815814	4	38816338	6.00E-13	12.222	Breast Neoplasms
HGVST2054	GWAS of Breast cancer	rs10022462	4	89243818	2.00E-09	8.699	Breast Neoplasms
HGVST2355	GWAS of Blood metabolites	rs10022462	4	89243818	5.00E-11	10.301	Blood
HGVST1616	GWAS of Breast cancer	rs9790517	4	1.06E+08	4.00E-08	7.398	Breast Neoplasms
HGVST2054	GWAS of Breast cancer	rs9790517	4	1.06E+08	5.00E-11	10.301	Breast Neoplasms
HGVST2746	GWAS of Breast cancer	rs9790517	4	1.06E+08	1.00E-09	9	Breast Neoplasms
HGVST2054	GWAS of Breast cancer	rs77528541	4	1.27E+08	1.00E-09	9	Breast Neoplasms
HGVST1616	GWAS of Breast cancer	rs6828523	4	1.76E+08	4.00E-16	15.398	Breast Neoplasms
HGVST2054	GWAS of Breast cancer	rs6828523	4	1.76E+08	2.00E-25	24.699	Breast Neoplasms
HGVST2746	GWAS of Breast cancer	rs6828523	4	1.76E+08	1.00E-15	15	Breast Neoplasms
HGVST2054	GWAS of Breast cancer	rs116095464	5	345109	4.00E-09	8.398	Breast Neoplasms
HGVST4003	GWAS of Autoimmune diseases and non-Hodg	rs10069690	5	1279790	5.00E-09	8.301	Lupus Erythematosus, Systemic
HGVST4003	GWAS of Autoimmune diseases and non-Hodg	rs10069690	5	1279790	5.00E-09	8.301	Leukemia, Lymphocytic, Chronic, B-Cell
HGVST935	GWAS of breast cancer	rs10069690	5	1279790	1.00E-10	10	Breast Neoplasms
HGVST1616	GWAS of Breast cancer	rs10069690	5	1279790	7.00E-09	8.155	Breast Neoplasms
HGVST1622	GWAS of Breast cancer	rs10069690	5	1279790	5.00E-12	11.301	Breast Neoplasms
HGVST2004	GWAS of Glioma	rs10069690	5	1279790	2.00E-06	5.699	Glioma
HGVST2004	GWAS of Glioma	rs10069690	5	1279790	8.00E-31	30.097	Glioma
HGVST2004	GWAS of Glioma	rs10069690	5	1279790	3.00E-35	34.523	Glioma
HGVST2054	GWAS of Breast cancer	rs10069690	5	1279790	8.00E-17	16.097	Breast Neoplasms
HGVST2318	GWAS of Breast cancer	rs10069690	5	1279790	1.00E-07	7	Triple Negative Breast Neoplasms
HGVST2402	GWAS of Epithelial ovarian cancer	rs10069690	5	1279790	9.00E-09	8.046	Ovarian Neoplasms
HGVST2403	GWAS of Breast cancer	rs10069690	5	1279790	2.00E-35	34.699	Breast Neoplasms
HGVST2403	GWAS of Breast cancer	rs10069690	5	1279790	4.00E-16	15.398	Breast Neoplasms
HGVST2424	GWAS of Thyroid cancer	rs10069690	5	1279790	3.00E-07	6.523	Thyroid Neoplasms
HGVST2600	GWAS of Glioma	rs10069690	5	1279790	4.00E-20	19.398	Central Nervous System Neoplasms
HGVST2600	GWAS of Glioma	rs10069690	5	1279790	8.00E-07	6.097	Central Nervous System Neoplasms
HGVST2746	GWAS of Breast cancer	rs10069690	5	1279790	1.00E-08	8	Breast Neoplasms
HGVST2831	GWAS of Epithelial ovarian cancer	rs10069690	5	1279790	3.00E-08	7.523	Ovarian Neoplasms
HGVST2831	GWAS of Epithelial ovarian cancer	rs10069690	5	1279790	4.00E-09	8.398	Ovarian Neoplasms
HGVST2831	GWAS of Epithelial ovarian cancer	rs10069690	5	1279790	5.00E-12	11.301	Ovarian Neoplasms
HGVST2831	GWAS of Epithelial ovarian cancer	rs10069690	5	1279790	1.00E-07	7	Ovarian Neoplasms
HGVST2831	GWAS of Epithelial ovarian cancer	rs10069690	5	1279790	1.00E-09	9	Ovarian Neoplasms
HGVST2831	GWAS of Epithelial ovarian cancer	rs10069690	5	1279790	2.00E-12	11.699	Ovarian Neoplasms
HGVST3200	GWAS of Glioblastoma and non-glioblastoma t	rs10069690	5	1279790	3.00E-66	65.523	Glioma
HGVST3200	GWAS of Glioblastoma and non-glioblastoma t	rs10069690	5	1279790	1.00E-16	16	Glioma
HGVST3200	GWAS of Glioblastoma and non-glioblastoma t	rs10069690	5	1279790	8.00E-74	73.097	Glioblastoma
HGVST3268	GWAS of Breast cancer	rs10069690	5	1279790	2.00E-10	9.699	Breast Neoplasms
HGVST3268	GWAS of Breast cancer	rs10069690	5	1279790	5.00E-06	5.301	Breast Neoplasms
HGVST3468	GWAS of Various traits	rs10069690	5	1279790	3.00E-12	11.523	Blood Pressure
HGVST3505	GWAS of Glioblastoma	rs10069690	5	1279790	4.00E-33	32.398	Glioblastoma
HGVST3541	GWAS of Uterine leiomyoma	rs10069690	5	1279790	4.00E-18	17.398	Myofibroma
HGVST3614	GWAS of Blood pressure	rs10069690	5	1279790	4.00E-21	20.398	Blood Pressure
HGVST2054	GWAS of Breast cancer	rs3215401	5	1296255	1.00E-20	20	Breast Neoplasms
HGVST2403	GWAS of Breast cancer	rs3215401	5	1296255	6.00E-21	20.222	Breast Neoplasms
HGVST2403	GWAS of Breast cancer	rs3215401	5	1296255	3.00E-07	6.523	Breast Neoplasms
HGVST2054	GWAS of Breast cancer	rs13162653	5	16187528	5.00E-07	6.301	Breast Neoplasms
HGVST2746	GWAS of Breast cancer	rs13162653	5	16187528	1.00E-10	10	Breast Neoplasms
HGVST2054	GWAS of Breast cancer	rs2012709	5	32567732	1.00E-08	8	Breast Neoplasms
HGVST2746	GWAS of Breast cancer	rs2012709	5	32567732	6.00E-09	8.222	Breast Neoplasms
HGVST1616	GWAS of Breast cancer	rs10941679	5	44706498	2.00E-37	36.699	Breast Neoplasms
HGVST2054	GWAS of Breast cancer	rs10941679	5	44706498	6.00E-73	72.222	Breast Neoplasms
HGVST2746	GWAS of Breast cancer	rs10941679	5	44706498	5.00E-32	31.301	Breast Neoplasms
HGVST2054	GWAS of Breast cancer	rs72749841	5	49641645	7.00E-10	9.155	Breast Neoplasms
HGVST2054	GWAS of Breast cancer	rs35951924	5	50195109	1.00E-11	11	Breast Neoplasms
HGVST2054	GWAS of Breast cancer	rs62355902	5	56053723	7.00E-98	97.155	Breast Neoplasms
HGVST2403	GWAS of Breast cancer	rs62355902	5	56053723	2.00E-06	5.699	Breast Neoplasms
HGVST1616	GWAS of Breast cancer	rs10472076	5	58184061	3.00E-08	7.523	Breast Neoplasms
HGVST2054	GWAS of Breast cancer	rs10472076	5	58184061	1.00E-08	8	Breast Neoplasms
HGVST2746	GWAS of Breast cancer	rs10472076	5	58184061	2.00E-08	7.699	Breast Neoplasms
HGVST1616	GWAS of Breast cancer	rs1353747	5	58337481	3.00E-08	7.523	Breast Neoplasms
HGVST2054	GWAS of Breast cancer	rs1353747	5	58337481	4.00E-09	8.398	Breast Neoplasms
HGVST2746	GWAS of Breast cancer	rs1353747	5	58337481	3.00E-08	7.523	Breast Neoplasms
HGVST2054	GWAS of Breast cancer	rs7707921	5	81538046	2.00E-12	11.699	Breast Neoplasms
HGVST2746	GWAS of Breast cancer	rs7707921	5	81538046	5.00E-11	10.301	Breast Neoplasms
HGVST2054	GWAS of Breast cancer	rs10474352	5	90732225	5.00E-11	10.301	Breast Neoplasms
HGVST2447	GWAS of Breast cancer	rs10474352	5	90732225	2.00E-09	8.699	Breast Neoplasms
HGVST2054	GWAS of Breast cancer	rs6882649	5	1.11E+08	4.00E-09	8.398	Breast Neoplasms
HGVST2054	GWAS of Breast cancer	rs6596100	5	1.32E+08	8.00E-09	8.097	Breast Neoplasms
HGVST1616	GWAS of Breast cancer	rs1432679	5	1.58E+08	2.00E-14	13.699	Breast Neoplasms
HGVST1622	GWAS of Breast cancer	rs1432679	5	1.58E+08	7.00E-06	5.155	Breast Neoplasms
HGVST2054	GWAS of Breast cancer	rs1432679	5	1.58E+08	7.00E-31	30.155	Breast Neoplasms
HGVST2403	GWAS of Breast cancer	rs1432679	5	1.58E+08	1.00E-10	10	Breast Neoplasms
HGVST2746	GWAS of Breast cancer	rs1432679	5	1.58E+08	4.00E-16	15.398	Breast Neoplasms
HGVST3137	GWAS of Breast cancer	rs1432679	5	1.58E+08	2.00E-06	5.699	Breast Neoplasms
HGVST2054	GWAS of Breast cancer	rs4562056	5	1.7E+08	5.00E-10	9.301	Breast Neoplasms
HGVST1616	GWAS of Breast cancer	rs11242675	6	1318878	7.00E-09	8.155	Breast Neoplasms
HGVST2746	GWAS of Breast cancer	rs11242675	6	1318878	1.00E-07	7	Breast Neoplasms
HGVST3653	GWAS of Breast cancer	rs9348512	6	10456706	4.00E-08	7.398	Breast Neoplasms
HGVST1616	GWAS of Breast cancer	rs204247	6	13722523	8.00E-09	8.097	Breast Neoplasms
HGVST2054	GWAS of Breast cancer	rs204247	6	13722523	8.00E-13	12.097	Breast Neoplasms
HGVST2746	GWAS of Breast cancer	rs204247	6	13722523	4.00E-10	9.398	Breast Neoplasms
HGVST2054	GWAS of Breast cancer	rs3819405	6	16399557	2.00E-08	7.699	Breast Neoplasms
HGVST2054	GWAS of Breast cancer	rs2223621	6	20621238	3.00E-10	9.523	Breast Neoplasms
HGVST2054	GWAS of Breast cancer	rs71557345	6	26680698	4.00E-10	9.398	Breast Neoplasms
HGVST2054	GWAS of Breast cancer	rs9257408	6	28926220	7.00E-08	7.155	Breast Neoplasms
HGVST2746	GWAS of Breast cancer	rs9257408	6	28926220	5.00E-08	7.301	Breast Neoplasms
HGVST2054	GWAS of Breast cancer	rs12207986	6	81094287	2.00E-09	8.699	Breast Neoplasms
HGVST2054	GWAS of Breast cancer	rs17529111	6	82128386	1.00E-09	9	Breast Neoplasms
HGVST2403	GWAS of Breast cancer	rs17529111	6	82128386	2.00E-06	5.699	Breast Neoplasms
HGVST2746	GWAS of Breast cancer	rs17529111	6	82128386	2.00E-10	9.699	Breast Neoplasms
HGVST634	GWAS of height	rs6569648	6	1.3E+08	1.00E-21	21	Body Height
HGVST634	GWAS of height	rs6569648	6	1.3E+08	8.93E-12	11.04919718	Body Height
HGVST3848	GWAS of Height	rs6569648	6	1.3E+08	8.00E-10	9.097	Body Height
HGVST1903	GWAS of Hematological traits	rs6569648	6	1.3E+08	2.00E-10	9.699	Lymphocyte Count
HGVST2054	GWAS of Breast cancer	rs6569648	6	1.3E+08	3.00E-12	11.523	Breast Neoplasms
HGVST2078	GWAS of Body fat distribution	rs6569648	6	1.3E+08	7.00E-12	11.155	Hip
HGVST2403	GWAS of Breast cancer	rs6569648	6	1.3E+08	4.00E-08	7.398	Breast Neoplasms
HGVST2403	GWAS of Breast cancer	rs6569648	6	1.3E+08	8.00E-10	9.097	Breast Neoplasms
HGVST2486	GWAS of Body mass index	rs6569648	6	1.3E+08	2.00E-06	5.699	Body Mass Index
HGVST3468	GWAS of Various traits	rs6569648	6	1.3E+08	5.00E-09	8.301	Hair Color
HGVST3588	GWAS of Body mass index	rs6569648	6	1.3E+08	5.00E-12	11.301	Body Mass Index

HGVST1053	GWAS of Breast cancer	rs9485372	6	1.5E+08	4.00E-12	NA	Breast Neoplasms
HGVST2054	GWAS of Breast cancer	rs9485372	6	1.5E+08	4.00E-06	5.398	Breast Neoplasms
HGVST2054	GWAS of Breast cancer	rs3757322	6	1.52E+08	3.00E-41	40.523	Breast Neoplasms
HGVST2403	GWAS of Breast cancer	rs3757322	6	1.52E+08	3.00E-31	30.523	Breast Neoplasms
HGVST2403	GWAS of Breast cancer	rs3757322	6	1.52E+08	3.00E-12	11.523	Breast Neoplasms
HGVST2054	GWAS of Breast cancer	rs9397437	6	1.52E+08	5.00E-54	53.301	Breast Neoplasms
HGVST2403	GWAS of Breast cancer	rs9397437	6	1.52E+08	3.00E-40	39.523	Breast Neoplasms
HGVST2403	GWAS of Breast cancer	rs9397437	6	1.52E+08	6.00E-08	7.222	Breast Neoplasms
HGVST3303	GWAS of Various traits	rs9397437	6	1.52E+08	1.00E-11	11	Breast
HGVST3795	GWAS of Gynecology-related traits	rs9397437	6	1.52E+08	2.00E-14	13.699	Breast
HGVST2054	GWAS of Breast cancer	rs2747652	6	1.52E+08	1.00E-26	26	Breast Neoplasms
HGVST2403	GWAS of Breast cancer	rs2747652	6	1.52E+08	2.00E-18	17.699	Breast Neoplasms
HGVST2054	GWAS of Breast cancer	rs7971	7	21940960	2.00E-08	7.699	Breast Neoplasms
HGVST2054	GWAS of Breast cancer	rs17156577	7	28356889	4.00E-09	8.398	Breast Neoplasms
HGVST2054	GWAS of Breast cancer	rs6964587	7	91630620	9.00E-11	10.046	Breast Neoplasms
HGVST2746	GWAS of Breast cancer	rs6964587	7	91630620	5.00E-07	6.301	Breast Neoplasms
HGVST2054	GWAS of Breast cancer	rs17268829	7	94113799	5.00E-13	12.301	Breast Neoplasms
HGVST2054	GWAS of Breast cancer	rs71559437	7	1.02E+08	5.00E-12	11.301	Breast Neoplasms
HGVST2054	GWAS of Breast cancer	rs4593472	7	1.31E+08	2.00E-11	10.699	Breast Neoplasms
HGVST2746	GWAS of Breast cancer	rs4593472	7	1.31E+08	2.00E-09	8.699	Breast Neoplasms
HGVST2054	GWAS of Breast cancer	rs11977670	7	1.4E+08	1.00E-16	16	Breast Neoplasms
HGVST1616	GWAS of Breast cancer	rs720475	7	1.44E+08	7.00E-11	10.155	Breast Neoplasms
HGVST2054	GWAS of Breast cancer	rs720475	7	1.44E+08	1.00E-11	11	Breast Neoplasms
HGVST2746	GWAS of Breast cancer	rs720475	7	1.44E+08	4.00E-10	9.398	Breast Neoplasms
HGVST2054	GWAS of Breast cancer	rs66823261	8	170692	3.00E-07	6.523	Breast Neoplasms
HGVST2403	GWAS of Breast cancer	rs66823261	8	170692	6.00E-09	8.222	Breast Neoplasms
HGVST2403	GWAS of Breast cancer	rs66823261	8	170692	3.00E-08	7.523	Breast Neoplasms
HGVST1616	GWAS of Breast cancer	rs9693444	8	29509616	9.00E-14	13.046	Breast Neoplasms
HGVST2054	GWAS of Breast cancer	rs9693444	8	29509616	2.00E-21	20.699	Breast Neoplasms
HGVST2746	GWAS of Breast cancer	rs9693444	8	29509616	1.00E-12	12	Breast Neoplasms
HGVST2054	GWAS of Breast cancer	rs13365225	8	36858483	1.00E-20	20	Breast Neoplasms
HGVST2403	GWAS of Breast cancer	rs13365225	8	36858483	1.00E-10	10	Breast Neoplasms
HGVST2746	GWAS of Breast cancer	rs13365225	8	36858483	1.00E-08	8	Breast Neoplasms
HGVST3137	GWAS of Breast cancer	rs13365225	8	36858483	7.00E-06	5.155	Breast Neoplasms
HGVST1616	GWAS of Breast cancer	rs6472903	8	76230301	2.00E-17	16.699	Breast Neoplasms
HGVST2054	GWAS of Breast cancer	rs6472903	8	76230301	4.00E-21	20.398	Breast Neoplasms
HGVST2746	GWAS of Breast cancer	rs6472903	8	76230301	1.00E-16	16	Breast Neoplasms
HGVST1616	GWAS of Breast cancer	rs2943559	8	76417937	6.00E-15	14.222	Breast Neoplasms
HGVST2054	GWAS of Breast cancer	rs2943559	8	76417937	4.00E-24	23.398	Breast Neoplasms
HGVST2403	GWAS of Breast cancer	rs2943559	8	76417937	3.00E-06	5.523	Breast Neoplasms
HGVST2746	GWAS of Breast cancer	rs2943559	8	76417937	1.00E-16	16	Breast Neoplasms
HGVST2054	GWAS of Breast cancer	rs514192	8	1.02E+08	6.00E-09	8.222	Breast Neoplasms
HGVST2054	GWAS of Breast cancer	rs12546444	8	1.06E+08	8.00E-11	10.097	Breast Neoplasms
HGVST2054	GWAS of Breast cancer	rs13267382	8	1.17E+08	2.00E-11	10.699	Breast Neoplasms
HGVST2746	GWAS of Breast cancer	rs13267382	8	1.17E+08	2.00E-08	7.699	Breast Neoplasms
HGVST2054	GWAS of Breast cancer	rs58847541	8	1.25E+08	6.00E-13	12.222	Breast Neoplasms
HGVST2403	GWAS of Breast cancer	rs17350191	8	1.25E+08	2.00E-08	7.699	Breast Neoplasms
HGVST2403	GWAS of Breast cancer	rs17350191	8	1.25E+08	2.00E-11	10.699	Breast Neoplasms
HGVST63	GWAS of breast cancer	rs13281615	8	1.28E+08	4.00E-12	11.39794001	Breast Neoplasms
HGVST1616	GWAS of Breast cancer	rs13281615	8	1.28E+08	1.00E-27	27	Breast Neoplasms
HGVST2054	GWAS of Breast cancer	rs13281615	8	1.28E+08	2.00E-57	56.699	Breast Neoplasms
HGVST2403	GWAS of Breast cancer	rs13281615	8	1.28E+08	9.00E-06	5.046	Breast Neoplasms
HGVST2746	GWAS of Breast cancer	rs13281615	8	1.28E+08	6.00E-31	30.222	Breast Neoplasms
HGVST1616	GWAS of Breast cancer	rs11780156	8	1.29E+08	3.00E-11	10.523	Breast Neoplasms
HGVST2054	GWAS of Breast cancer	rs11780156	8	1.29E+08	1.00E-13	13	Breast Neoplasms
HGVST2746	GWAS of Breast cancer	rs11780156	8	1.29E+08	2.00E-09	8.699	Breast Neoplasms
HGVST532	GWAS of breast cancer	rs1011970	9	22062134	3.00E-08	7.522878745	Breast Neoplasms
HGVST1616	GWAS of Breast cancer	rs1011970	9	22062134	6.00E-08	7.222	Breast Neoplasms
HGVST2054	GWAS of Breast cancer	rs1011970	9	22062134	1.00E-15	15	Breast Neoplasms
HGVST2320	GWAS of Cancer	rs1011970	9	22062134	2.00E-07	6.699	Neoplasms
HGVST2320	GWAS of Cancer	rs1011970	9	22062134	5.00E-06	5.301	Neoplasms
HGVST2403	GWAS of Breast cancer	rs1011970	9	22062134	5.00E-07	6.301	Breast Neoplasms
HGVST2746	GWAS of Breast cancer	rs1011970	9	22062134	4.00E-09	8.398	Breast Neoplasms
HGVST3137	GWAS of Breast cancer	rs1011970	9	22062134	2.00E-06	5.699	Breast Neoplasms
HGVST1616	GWAS of Breast cancer	rs10759243	9	1.1E+08	1.00E-08	8	Breast Neoplasms
HGVST2054	GWAS of Breast cancer	rs10759243	9	1.1E+08	2.00E-18	17.699	Breast Neoplasms
HGVST2746	GWAS of Breast cancer	rs10759243	9	1.1E+08	3.00E-09	8.523	Breast Neoplasms
HGVST2054	GWAS of Breast cancer	rs10816625	9	1.11E+08	5.00E-18	17.301	Breast Neoplasms
HGVST2054	GWAS of Breast cancer	rs13294895	9	1.11E+08	7.00E-17	16.155	Breast Neoplasms
HGVST2054	GWAS of Breast cancer	rs676256	9	1.11E+08	4.00E-53	52.398	Breast Neoplasms
HGVST2054	GWAS of Breast cancer	rs1895062	9	1.19E+08	1.00E-14	14	Breast Neoplasms
HGVST2054	GWAS of Breast cancer	rs10760444	9	1.29E+08	9.00E-09	8.046	Breast Neoplasms
HGVST2054	GWAS of Breast cancer	rs8176636	9	1.36E+08	1.00E-08	8	Breast Neoplasms
HGVST532	GWAS of breast cancer	rs2380205	10	5886734	5.00E-07	6.301029996	Breast Neoplasms
HGVST2054	GWAS of Breast cancer	rs67958007	10	9088114	2.00E-10	9.699	Breast Neoplasms
HGVST3953	GWAS of Urinary sodium and potassium excret	rs7072776	10	22032942	7.00E-14	13.155	Sodium
HGVST1616	GWAS of Breast cancer	rs7072776	10	22032942	4.00E-14	13.398	Breast Neoplasms
HGVST2054	GWAS of Breast cancer	rs7072776	10	22032942	2.00E-19	18.699	Breast Neoplasms
HGVST2746	GWAS of Breast cancer	rs7072776	10	22032942	1.00E-14	14	Breast Neoplasms
HGVST3467	GWAS of Tobacco and alcohol use	rs7072776	10	22032942	3.00E-18	17.523	Smoking
HGVST3467	GWAS of Tobacco and alcohol use	rs7072776	10	22032942	1.00E-09	9	Smoking
HGVST3467	GWAS of Tobacco and alcohol use	rs7072776	10	22032942	6.00E-15	14.222	Smoking
HGVST1616	GWAS of Breast cancer	rs11814448	10	22315843	9.00E-16	15.046	Breast Neoplasms
HGVST2054	GWAS of Breast cancer	rs11814448	10	22315843	6.00E-18	17.222	Breast Neoplasms
HGVST2746	GWAS of Breast cancer	rs11814448	10	22315843	6.00E-17	16.222	Breast Neoplasms
HGVST2054	GWAS of Breast cancer	rs10995201	10	64299890	2.00E-51	50.699	Breast Neoplasms
HGVST2403	GWAS of Breast cancer	rs10995201	10	64299890	2.00E-09	8.699	Breast Neoplasms
HGVST532	GWAS of breast cancer	rs704010	10	80841148	4.00E-09	8.397940009	Breast Neoplasms
HGVST1616	GWAS of Breast cancer	rs704010	10	80841148	7.00E-22	21.155	Breast Neoplasms
HGVST2054	GWAS of Breast cancer	rs704010	10	80841148	2.00E-35	34.699	Breast Neoplasms
HGVST2746	GWAS of Breast cancer	rs704010	10	80841148	3.00E-23	22.523	Breast Neoplasms
HGVST2054	GWAS of Breast cancer	rs140936696	10	95292205	4.00E-08	7.398	Breast Neoplasms
HGVST17	GWAS of type II diabetes mellitus	rs7904519	10	1.15E+08	2.70E-10	9.568636236	Diabetes Mellitus, Type 2
HGVST54	GWAS of type II diabetes mellitus	rs7904519	10	1.15E+08	2.60E-06	5.585026652	Diabetes Mellitus, Type 2
HGVST907	GWAS of proinsulin levels	rs7904519	10	1.15E+08	3.34E-11	10.476	Proinsulin
HGVST1616	GWAS of Breast cancer	rs7904519	10	1.15E+08	3.00E-08	7.523	Breast Neoplasms
HGVST2054	GWAS of Breast cancer	rs7904519	10	1.15E+08	2.00E-13	12.699	Breast Neoplasms
HGVST2403	GWAS of Breast cancer	rs7904519	10	1.15E+08	8.00E-10	9.097	Breast Neoplasms
HGVST2746	GWAS of Breast cancer	rs7904519	10	1.15E+08	9.00E-13	12.046	Breast Neoplasms
HGVST1616	GWAS of Breast cancer	rs11199914	10	1.23E+08	2.00E-08	7.699	Breast Neoplasms
HGVST2054	GWAS of Breast cancer	rs11199914	10	1.23E+08	7.00E-12	11.155	Breast Neoplasms
HGVST2746	GWAS of Breast cancer	rs11199914	10	1.23E+08	8.00E-09	8.097	Breast Neoplasms
HGVST63	GWAS of breast cancer	rs2981578	10	1.23E+08	1.00E-15	15	Breast Neoplasms

HGVST1765	GWAS of Breast cancer	rs2981578	10	1.23E+08	1.00E-12	12	Breast Neoplasms
HGVST2054	GWAS of Breast cancer	rs2981578	10	1.23E+08	1.00E-245	245	Breast Neoplasms
HGVST2054	GWAS of Breast cancer	rs45631563	10	1.23E+08	7.00E-37	36.155	Breast Neoplasms
HGVST2054	GWAS of Breast cancer	rs6597981	11	803017	1.00E-12	12	Breast Neoplasms
HGVST63	GWAS of Breast cancer	rs3817198	11	1909006	3.00E-09	8.522878745	Breast Neoplasms
HGVST1616	GWAS of Breast cancer	rs3817198	11	1909006	2.00E-11	10.699	Breast Neoplasms
HGVST2054	GWAS of Breast cancer	rs3817198	11	1909006	1.00E-18	18	Breast Neoplasms
HGVST2746	GWAS of Breast cancer	rs3817198	11	1909006	2.00E-13	12.699	Breast Neoplasms
HGVST3253	GWAS of Mammographic density	rs3817198	11	1909006	1.00E-10	10	Breast Density
HGVST1616	GWAS of Breast cancer	rs3903072	11	65583066	9.00E-12	11.046	Breast Neoplasms
HGVST2054	GWAS of Breast cancer	rs3903072	11	65583066	2.00E-12	11.699	Breast Neoplasms
HGVST2746	GWAS of Breast cancer	rs3903072	11	65583066	1.00E-10	10	Breast Neoplasms
HGVST2054	GWAS of Breast cancer	rs554219	11	69331642	6.00E-47	46.222	Breast Neoplasms
HGVST2746	GWAS of Breast cancer	rs554219	11	69331642	2.00E-81	80.699	Breast Neoplasms
HGVST2054	GWAS of Breast cancer	rs75915166	11	69379161	4.00E-95	94.398	Breast Neoplasms
HGVST2746	GWAS of Breast cancer	rs75915166	11	69379161	1.00E-57	57	Breast Neoplasms
HGVST2403	GWAS of Breast cancer	rs11374964	11	1.08E+08	4.00E-08	7.398	Breast Neoplasms
HGVST2403	GWAS of Breast cancer	rs11374964	11	1.08E+08	1.00E-06	6	Breast Neoplasms
HGVST2403	GWAS of Breast cancer	rs11374964	11	1.08E+08	4.00E-13	12.398	Breast Neoplasms
HGVST2253	GWAS of Blood protein levels	rs74911261	11	1.08E+08	1.00E-69	69	Blood Proteins
HGVST2367	GWAS of Renal cell carcinoma	rs74911261	11	1.08E+08	2.00E-10	9.699	Carcinoma, Renal Cell
HGVST2403	GWAS of Breast cancer	rs74911261	11	1.08E+08	2.00E-06	5.699	Breast Neoplasms
HGVST2403	GWAS of Breast cancer	rs74911261	11	1.08E+08	2.00E-06	5.699	Breast Neoplasms
HGVST2403	GWAS of Breast cancer	rs74911261	11	1.08E+08	5.00E-11	10.301	Breast Neoplasms
HGVST3734	GWAS of Blood proteins	rs74911261	11	1.08E+08	1.00E-68	68	Blood Proteins
HGVST1616	GWAS of Breast cancer	rs11820646	11	1.29E+08	1.00E-09	9	Breast Neoplasms
HGVST2054	GWAS of Breast cancer	rs11820646	11	1.29E+08	2.00E-14	13.699	Breast Neoplasms
HGVST2403	GWAS of Breast cancer	rs11820646	11	1.29E+08	2.00E-07	6.699	Breast Neoplasms
HGVST2746	GWAS of Breast cancer	rs11820646	11	1.29E+08	1.00E-10	10	Breast Neoplasms
HGVST3137	GWAS of Breast cancer	rs11820646	11	1.29E+08	9.00E-06	5.046	Breast Neoplasms
HGVST1616	GWAS of Breast cancer	rs12422552	12	14413931	4.00E-08	7.398	Breast Neoplasms
HGVST2054	GWAS of Breast cancer	rs12422552	12	14413931	4.00E-15	14.398	Breast Neoplasms
HGVST2746	GWAS of Breast cancer	rs12422552	12	14413931	1.00E-07	7	Breast Neoplasms
HGVST3468	GWAS of Various traits	rs12422552	12	14413931	1.00E-09	9	Body Mass Index
HGVST2054	GWAS of Breast cancer	rs7297051	12	28174817	3.00E-60	59.523	Breast Neoplasms
HGVST2403	GWAS of Breast cancer	rs7297051	12	28174817	9.00E-25	24.046	Breast Neoplasms
HGVST2403	GWAS of Breast cancer	rs7297051	12	28174817	8.00E-09	8.097	Breast Neoplasms
HGVST3137	GWAS of Breast cancer	rs7297051	12	28174817	1.00E-14	14	Breast Neoplasms
HGVST3137	GWAS of Breast cancer	rs7297051	12	28174817	3.00E-20	19.523	Breast Neoplasms
HGVST2054	GWAS of Breast cancer	rs202049448	12	85009437	3.00E-08	7.523	Breast Neoplasms
HGVST1353	GWAS of Breast size	rs17356907	12	96027759	1.00E-06	6	Breast Neoplasms
HGVST1353	GWAS of Breast size	rs17356907	12	96027759	1.00E-06	6	Breast
HGVST1616	GWAS of Breast cancer	rs17356907	12	96027759	2.00E-22	21.699	Breast Neoplasms
HGVST1622	GWAS of Breast cancer	rs17356907	12	96027759	9.00E-06	5.046	Breast Neoplasms
HGVST2054	GWAS of Breast cancer	rs17356907	12	96027759	1.00E-39	39	Breast Neoplasms
HGVST2403	GWAS of Breast cancer	rs17356907	12	96027759	2.00E-08	7.699	Breast Neoplasms
HGVST2746	GWAS of Breast cancer	rs17356907	12	96027759	7.00E-21	20.155	Breast Neoplasms
HGVST3137	GWAS of Breast cancer	rs17356907	12	96027759	3.00E-06	5.523	Breast Neoplasms
HGVST3137	GWAS of Breast cancer	rs17356907	12	96027759	5.00E-07	6.301	Breast Neoplasms
HGVST3303	GWAS of Various traits	rs17356907	12	96027759	1.00E-13	13	Breast
HGVST1616	GWAS of Breast cancer	rs1292011	12	1.16E+08	9.00E-22	21.046	Breast Neoplasms
HGVST2054	GWAS of Breast cancer	rs1292011	12	1.16E+08	4.00E-39	38.398	Breast Neoplasms
HGVST2746	GWAS of Breast cancer	rs1292011	12	1.16E+08	2.00E-21	20.699	Breast Neoplasms
HGVST2054	GWAS of Breast cancer	rs206966	12	1.21E+08	4.00E-08	7.398	Breast Neoplasms
HGVST1616	GWAS of Breast cancer	rs11571833	13	32972626	5.00E-08	7.301	Breast Neoplasms
HGVST1622	GWAS of Breast cancer	rs11571833	13	32972626	6.00E-06	5.222	Breast Neoplasms
HGVST2054	GWAS of Breast cancer	rs11571833	13	32972626	3.00E-15	14.523	Breast Neoplasms
HGVST2099	GWAS of Lung cancer	rs11571833	13	32972626	3.00E-08	7.523	Small Cell Lung Carcinoma
HGVST2099	GWAS of Lung cancer	rs11571833	13	32972626	6.00E-16	15.222	Lung Neoplasms
HGVST2099	GWAS of Lung cancer	rs11571833	13	32972626	6.00E-08	7.222	Lung Neoplasms
HGVST2320	GWAS of Cancer	rs11571833	13	32972626	8.00E-12	11.097	Neoplasms
HGVST2403	GWAS of Breast cancer	rs11571833	13	32972626	2.00E-13	12.699	Breast Neoplasms
HGVST2703	GWAS of Lung cancer	rs11571833	13	32972626	5.00E-20	19.301	Lung Neoplasms
HGVST2746	GWAS of Breast cancer	rs11571833	13	32972626	6.00E-07	6.222	Breast Neoplasms
HGVST3137	GWAS of Breast cancer	rs11571833	13	32972626	6.00E-06	5.222	Breast Neoplasms
HGVST2054	GWAS of Breast cancer	rs6562760	13	73957681	2.00E-09	8.699	Breast Neoplasms
HGVST2403	GWAS of Breast cancer	rs6562760	13	73957681	9.00E-10	9.046	Breast Neoplasms
HGVST2403	GWAS of Breast cancer	rs6562760	13	73957681	4.00E-07	6.398	Breast Neoplasms
HGVST3137	GWAS of Breast cancer	rs6562760	13	73957681	5.00E-10	9.301	Breast Neoplasms
HGVST1616	GWAS of Breast cancer	rs2236007	14	37132769	2.00E-13	12.699	Breast Neoplasms
HGVST2054	GWAS of Breast cancer	rs2236007	14	37132769	4.00E-21	20.398	Breast Neoplasms
HGVST2746	GWAS of Breast cancer	rs2236007	14	37132769	9.00E-13	12.046	Breast Neoplasms
HGVST634	GWAS of height	rs2588809	14	68660428	7.36E-06	5.133181197	Body Height
HGVST1616	GWAS of Breast cancer	rs2588809	14	68660428	1.00E-10	10	Breast Neoplasms
HGVST2054	GWAS of Breast cancer	rs2588809	14	68660428	6.00E-14	13.222	Breast Neoplasms
HGVST2746	GWAS of Breast cancer	rs2588809	14	68660428	2.00E-08	7.699	Breast Neoplasms
HGVST155	GWAS of breast cancer	rs999737	14	69034682	2.00E-07	6.698970004	Breast Neoplasms
HGVST1616	GWAS of Breast cancer	rs999737	14	69034682	3.00E-19	18.523	Breast Neoplasms
HGVST2054	GWAS of Breast cancer	rs999737	14	69034682	7.00E-39	38.155	Breast Neoplasms
HGVST2403	GWAS of Breast cancer	rs999737	14	69034682	3.00E-08	7.523	Breast Neoplasms
HGVST2746	GWAS of Breast cancer	rs999737	14	69034682	2.00E-21	20.699	Breast Neoplasms
HGVST1616	GWAS of Breast cancer	rs941764	14	91841069	4.00E-10	9.398	Breast Neoplasms
HGVST2054	GWAS of Breast cancer	rs941764	14	91841069	8.00E-13	12.097	Breast Neoplasms
HGVST2746	GWAS of Breast cancer	rs941764	14	91841069	6.00E-11	10.222	Breast Neoplasms
HGVST2054	GWAS of Breast cancer	rs11627032	14	93104072	4.00E-11	10.398	Breast Neoplasms
HGVST2403	GWAS of Breast cancer	rs11627032	14	93104072	2.00E-06	5.699	Breast Neoplasms
HGVST2746	GWAS of Breast cancer	rs11627032	14	93104072	4.00E-09	8.398	Breast Neoplasms
HGVST2054	GWAS of Breast cancer	rs10623258	14	1.05E+08	2.00E-08	7.699	Breast Neoplasms
HGVST2054	GWAS of Breast cancer	rs2290203	15	91512067	8.00E-10	9.097	Breast Neoplasms
HGVST2447	GWAS of Breast cancer	rs2290203	15	91512067	4.00E-08	7.398	Breast Neoplasms
HGVST3596	GWAS of Type 2 diabetes	rs2290203	15	91512067	2.00E-10	9.699	Diabetes Mellitus, Type 2
HGVST2403	GWAS of Breast cancer	rs11076805	16	4106788	2.00E-08	7.699	Breast Neoplasms
HGVST2403	GWAS of Breast cancer	rs11076805	16	4106788	1.00E-08	8	Breast Neoplasms
HGVST530	GWAS of breast cancer	rs4784227	16	52599188	1.00E-28	28	Breast Neoplasms
HGVST2054	GWAS of Breast cancer	rs4784227	16	52599188	7.00E-201	200.155	Breast Neoplasms
HGVST2154	GWAS of Parkinson's disease	rs4784227	16	52599188	1.00E-10	10	Parkinson Disease
HGVST2403	GWAS of Breast cancer	rs4784227	16	52599188	2.00E-26	25.699	Breast Neoplasms
HGVST2640	GWAS of Breast cancer	rs4784227	16	52599188	3.00E-09	8.523	Breast Neoplasms
HGVST3137	GWAS of Breast cancer	rs4784227	16	52599188	1.00E-14	14	Breast Neoplasms
HGVST3137	GWAS of Breast cancer	rs4784227	16	52599188	6.00E-15	14.222	Breast Neoplasms
HGVST3429	GWAS of BRCA1/2-negative high-risk breast ca	rs4784227	16	52599188	2.00E-09	8.699	Breast Neoplasms
HGVST3468	GWAS of Various traits	rs4784227	16	52599188	2.00E-08	7.699	Blood Pressure
HGVST3710	GWAS of Breast cancer	rs4784227	16	52599188	7.00E-06	5.155	Breast Neoplasms

HGVST640	GWAS of body mass index	rs17817449	16	53813367	3.67E-60	59.435	Body Mass Index
HGVST871	GWAS of obesity	rs17817449	16	53813367	2.00E-12	11.69897	Obesity
HGVST1616	GWAS of Breast cancer	rs17817449	16	53813367	6.00E-14	13.222	Breast Neoplasms
HGVST1622	GWAS of Breast cancer	rs17817449	16	53813367	6.00E-07	6.222	Breast Neoplasms
HGVST2054	GWAS of Breast cancer	rs17817449	16	53813367	3.00E-21	20.523	Breast Neoplasms
HGVST2403	GWAS of Breast cancer	rs17817449	16	53813367	2.00E-10	9.699	Breast Neoplasms
HGVST2746	GWAS of Breast cancer	rs17817449	16	53813367	2.00E-14	13.699	Breast Neoplasms
HGVST3137	GWAS of Breast cancer	rs17817449	16	53813367	3.00E-07	6.523	Breast Neoplasms
HGVST3137	GWAS of Breast cancer	rs17817449	16	53813367	5.00E-08	7.301	Breast Neoplasms
HGVST3327	GWAS of Various traits	rs17817449	16	53813367	5.00E-19	18.301	Body Mass Index
HGVST640	GWAS of body mass index	rs11075995	16	53855291	1.51E-06	5.821	Body Mass Index
HGVST1622	GWAS of Breast cancer	rs11075995	16	53855291	4.00E-08	7.398	Breast Neoplasms
HGVST2054	GWAS of Breast cancer	rs11075995	16	53855291	9.00E-09	8.046	Breast Neoplasms
HGVST2403	GWAS of Breast cancer	rs11075995	16	53855291	1.00E-10	10	Breast Neoplasms
HGVST2746	GWAS of Breast cancer	rs11075995	16	53855291	1.00E-06	6	Breast Neoplasms
HGVST3137	GWAS of Breast cancer	rs11075995	16	53855291	3.00E-08	7.523	Breast Neoplasms
HGVST3137	GWAS of Breast cancer	rs11075995	16	53855291	2.00E-06	5.699	Breast Neoplasms
HGVST2054	GWAS of Breast cancer	rs28539243	16	54682064	9.00E-15	14.046	Breast Neoplasms
HGVST2054	GWAS of Breast cancer	rs2432539	16	56420987	4.00E-08	7.398	Breast Neoplasms
HGVST1616	GWAS of Breast cancer	rs13329835	16	80650805	2.00E-16	15.699	Breast Neoplasms
HGVST2054	GWAS of Breast cancer	rs13329835	16	80650805	9.00E-27	26.046	Breast Neoplasms
HGVST2746	GWAS of Breast cancer	rs13329835	16	80650805	2.00E-17	16.699	Breast Neoplasms
HGVST2054	GWAS of Breast cancer	rs4496150	16	87085237	8.00E-09	8.097	Breast Neoplasms
HGVST2054	GWAS of Breast cancer	rs146699004	17	29230521	2.00E-09	8.699	Breast Neoplasms
HGVST2054	GWAS of Breast cancer	rs72826962	17	40836389	5.00E-09	8.301	Breast Neoplasms
HGVST2054	GWAS of Breast cancer	rs2532263	17	44252468	7.00E-13	12.155	Breast Neoplasms
HGVST3508	GWAS of General factor of neuroticism	rs2532263	17	44252468	4.00E-20	19.398	Neuroticism
HGVST2054	GWAS of Breast cancer	rs2787486	17	53209774	6.00E-29	28.222	Breast Neoplasms
HGVST2054	GWAS of Breast cancer	rs745570	17	77781725	4.00E-10	9.398	Breast Neoplasms
HGVST2746	GWAS of Breast cancer	rs745570	17	77781725	1.00E-09	9	Breast Neoplasms
HGVST3137	GWAS of Breast cancer	rs745570	17	77781725	6.00E-06	5.222	Breast Neoplasms
HGVST3467	GWAS of Tobacco and alcohol use	rs745570	17	77781725	3.00E-10	9.523	Smoking
HGVST3740	GWAS of Risk tolerance and risky behaviors	rs745570	17	77781725	1.00E-08	8	Smoking
HGVST1616	GWAS of Breast cancer	rs527616	18	24337424	2.00E-10	9.699	Breast Neoplasms
HGVST2054	GWAS of Breast cancer	rs527616	18	24337424	7.00E-15	14.155	Breast Neoplasms
HGVST2746	GWAS of Breast cancer	rs527616	18	24337424	8.00E-13	12.097	Breast Neoplasms
HGVST1616	GWAS of Breast cancer	rs1436904	18	24570667	3.00E-08	7.523	Breast Neoplasms
HGVST2054	GWAS of Breast cancer	rs1436904	18	24570667	1.00E-14	14	Breast Neoplasms
HGVST2746	GWAS of Breast cancer	rs1436904	18	24570667	3.00E-08	7.523	Breast Neoplasms
HGVST2403	GWAS of Breast cancer	rs36194942	18	25401205	3.00E-07	6.523	Breast Neoplasms
HGVST2403	GWAS of Breast cancer	rs36194942	18	25401205	1.00E-08	8	Breast Neoplasms
HGVST2054	GWAS of Breast cancer	rs117618124	18	29977689	6.00E-12	11.222	Breast Neoplasms
HGVST2403	GWAS of Breast cancer	rs117618124	18	29977689	5.00E-06	5.301	Breast Neoplasms
HGVST2054	GWAS of Breast cancer	rs6507583	18	42399590	2.00E-12	11.699	Breast Neoplasms
HGVST2746	GWAS of Breast cancer	rs6507583	18	42399590	3.00E-08	7.523	Breast Neoplasms
HGVST2403	GWAS of Breast cancer	rs322144	19	11423703	7.00E-09	8.155	Breast Neoplasms
HGVST2054	GWAS of Breast cancer	rs78269692	19	13158277	2.00E-09	8.699	Breast Neoplasms
HGVST2054	GWAS of Breast cancer	rs2594714	19	13954571	1.00E-08	8	Breast Neoplasms
HGVST2054	GWAS of Breast cancer	rs67397200	19	17401404	2.00E-08	7.699	Breast Neoplasms
HGVST2403	GWAS of Breast cancer	rs67397200	19	17401404	3.00E-37	36.523	Breast Neoplasms
HGVST2403	GWAS of Breast cancer	rs67397200	19	17401404	3.00E-17	16.523	Breast Neoplasms
HGVST1616	GWAS of Breast cancer	rs4808801	19	18571141	5.00E-15	14.301	Breast Neoplasms
HGVST1827	GWAS of Metabolite quantitative traits	rs4808801	19	18571141	9.79E-06	5.009	Inositol
HGVST2054	GWAS of Breast cancer	rs4808801	19	18571141	5.00E-28	27.301	Breast Neoplasms
HGVST2403	GWAS of Breast cancer	rs4808801	19	18571141	6.00E-06	5.222	Breast Neoplasms
HGVST2746	GWAS of Breast cancer	rs4808801	19	18571141	4.00E-15	14.398	Breast Neoplasms
HGVST2054	GWAS of Breast cancer	rs2965183	19	19545696	6.00E-12	11.222	Breast Neoplasms
HGVST2403	GWAS of Breast cancer	rs113701136	19	30277729	2.00E-07	6.699	Breast Neoplasms
HGVST2403	GWAS of Breast cancer	rs113701136	19	30277729	7.00E-09	8.155	Breast Neoplasms
HGVST3468	GWAS of Various traits	rs113701136	19	30277729	2.00E-24	23.699	Body Mass Index
HGVST3651	GWAS of Medication use	rs113701136	19	30277729	3.00E-10	9.523	Diuretics
HGVST1616	GWAS of Breast cancer	rs3760982	19	44286513	2.00E-10	9.699	Breast Neoplasms
HGVST2054	GWAS of Breast cancer	rs3760982	19	44286513	1.00E-16	16	Breast Neoplasms
HGVST2403	GWAS of Breast cancer	rs3760982	19	44286513	5.00E-08	7.301	Breast Neoplasms
HGVST2746	GWAS of Breast cancer	rs3760982	19	44286513	8.00E-09	8.097	Breast Neoplasms
HGVST2054	GWAS of Breast cancer	rs71338792	19	46183031	4.00E-09	8.398	Breast Neoplasms
HGVST274	GWAS of age at menarche and age at natural n	rs16991615	20	5948227	1.00E-21	21	Menarche
HGVST274	GWAS of age at menarche and age at natural n	rs16991615	20	5948227	1.00E-21	21	Menopause
HGVST1038	GWAS of Menopause (age at onset)	rs16991615	20	5948227	1.00E-73	NA	Menopause
HGVST1553	GWAS of Menopause (age at onset)	rs16991615	20	5948227	5.00E-12	11.301	Menopause
HGVST2054	GWAS of Breast cancer	rs16991615	20	5948227	2.00E-09	8.699	Breast Neoplasms
HGVST2552	GWAS of Menopause	rs16991615	20	5948227	2.00E-89	88.699	Menopause
HGVST3468	GWAS of Various traits	rs16991615	20	5948227	1.00E-143	143	Menopause
HGVST3482	GWAS of Anti-Mullerian hormone levels	rs16991615	20	5948227	4.00E-10	9.398	Anti-Mullerian Hormone
HGVST3541	GWAS of Uterine leiomyoma	rs16991615	20	5948227	2.00E-10	9.699	Myofibroma
HGVST1420	GWAS of Breast cancer	rs2284378	20	32588095	1.00E-08	8	Breast Neoplasms
HGVST2054	GWAS of Breast cancer	rs6122906	20	48945911	3.00E-10	9.523	Breast Neoplasms
HGVST1616	GWAS of Breast cancer	rs2823093	21	16520832	7.00E-16	15.155	Breast Neoplasms
HGVST2054	GWAS of Breast cancer	rs2823093	21	16520832	2.00E-20	19.699	Breast Neoplasms
HGVST2746	GWAS of Breast cancer	rs2823093	21	16520832	3.00E-13	12.523	Breast Neoplasms
HGVST2054	GWAS of Breast cancer	rs17879961	22	29121087	1.00E-08	8	Breast Neoplasms
HGVST2703	GWAS of Lung cancer	rs17879961	22	29121087	1.00E-13	13	Lung Neoplasms
HGVST1616	GWAS of Breast cancer	rs132390	22	29621477	3.00E-09	8.523	Breast Neoplasms
HGVST2054	GWAS of Breast cancer	rs132390	22	29621477	1.00E-08	8	Breast Neoplasms
HGVST2746	GWAS of Breast cancer	rs132390	22	29621477	3.00E-09	8.523	Breast Neoplasms
HGVST2054	GWAS of Breast cancer	rs738321	22	38568833	1.00E-13	13	Breast Neoplasms
HGVST1616	GWAS of Breast cancer	rs6001930	22	40876234	9.00E-19	18.046	Breast Neoplasms
HGVST1622	GWAS of Breast cancer	rs6001930	22	40876234	2.00E-06	5.699	Breast Neoplasms
HGVST2054	GWAS of Breast cancer	rs6001930	22	40876234	4.00E-34	33.398	Breast Neoplasms
HGVST2403	GWAS of Breast cancer	rs6001930	22	40876234	2.00E-11	10.699	Breast Neoplasms
HGVST2746	GWAS of Breast cancer	rs6001930	22	40876234	2.00E-20	19.699	Breast Neoplasms
HGVST3137	GWAS of Breast cancer	rs6001930	22	40876234	2.00E-06	5.699	Breast Neoplasms
HGVST2054	GWAS of Breast cancer	rs73161324	22	42038786	2.00E-09	8.699	Breast Neoplasms
HGVST2068	GWAS of Blood pressure	rs73161324	22	42038786	3.00E-11	10.523	Blood Pressure
HGVST2054	GWAS of Breast cancer	rs28512361	22	46283297	2.00E-08	7.699	Breast Neoplasms
